# Breast Cancer Identification via Thermography Image Segmentation with a Gradient Vector Flow and a Convolutional Neural Network

**DOI:** 10.1155/2019/9807619

**Published:** 2019-11-03

**Authors:** Santiago Tello-Mijares, Fomuy Woo, Francisco Flores

**Affiliations:** ^1^Instituto Tecnológico Superior de Lerdo, Postgraduate Department, Lerdo 35150, Mexico; ^2^Instituto Tecnológico de la Laguna, Postgraduate Department, Torreón 27000, Mexico; ^3^Instituto de Seguridad y Servicios Sociales de los Trabajadores del Estado, Medical Familiar Unit, Torreón 27268, Mexico

## Abstract

Breast cancer is the most common cancer among women worldwide with about half a million cases reported each year. Mammary thermography can offer early diagnosis at low cost if adequate thermographic images of the breasts are taken. The identification of breast cancer in an automated way can accelerate many tasks and applications of pathology. This can help complement diagnosis. The aim of this work is to develop a system that automatically captures thermographic images of breast and classifies them as normal and abnormal (without cancer and with cancer). This paper focuses on a segmentation method based on a combination of the curvature function *k* and the gradient vector flow, and for classification, we proposed a convolutional neural network (CNN) using the segmented breast. The aim of this paper is to compare CNN results with other classification techniques. Thus, every breast is characterized by its shape, colour, and texture, as well as left or right breast. These data were used for training as well as to compare the performance of CNN with three classification techniques: tree random forest (TRF), multilayer perceptron (MLP), and Bayes network (BN). CNN presents better results than TRF, MLP, and BN.

## 1. Introduction

Breast cancer is the most common cancer worldwide among women; approximately 2 in 5 women worldwide will develop breast cancer during their lives [1]. Since 2013, breast cancer has been the leading cause of death in women [2]. The World Health Organization (WHO) estimates that by the year 2030, an estimated 27 million new cases can be expected [3]. Early detection of this disease plays an important role in reducing the mortality rate [4]; if the tumor is detected before reaching a size of 10 mm, the patient has an 85% chance of complete remission [5]. There are currently many different techniques to diagnose this pathology (mammography, ultrasound, magnetic resonance, biopsies, and more recently thermography) [6]. Mammography is currently the most common technique, but it uses ionizing radiation and is painful due to breast compression [5]. It detects cancer 8 to 10 years later than thermography [4].

In recent years, there has been a growing interest in the analysis of thermography images [7–10] to detect breast cancer. These techniques can increase productivity in the analysis of breast cancer and reduce detection errors [11]. Next, we summarize the principal computer vision works on the subject in recent years, segmentation and classification.

The first task in a computer vision system is segmentation. There are various examples of segmentation work [2, 7, 12–19]. In one study, the software package ThermoMED was used to investigate the ability of thermography to detect multicentric or multifocal breast carcinomas in a preoperative setting [12]. Breast thermogram images have been segmented using a projection profile approach and by asymmetry analysis of the left and right breasts to detect cancer [13]. Segmentation has also been done using by a Gaussian mixture model. The Gaussian mixture model parameters are estimated using the expectation-maximization algorithm [14]. Whole-body PET-CT and thermograms were compared in diagnosing breast cancer with breast biopsy as a standard [15].

A novel extended hidden Markov model (EHMM) was presented for optimized segmentation of breast thermograms and was compared with other segmentation techniques [7]. A blood vessel segmentation method was proposed using three enhanced images to detect possible vessel regions based on their intensity and shape [16]. A segmentation technique was proposed for thermographic images, which considers the spatial information of the pixel contained in the image [2].

Important advances in the field have been achieved in [17–19]. In one study, the tumor region was found by applying fuzzy *c*-means for segmentation of the hottest regions in abnormal breasts [17]. Three image segmentation methods, *k*-means, fuzzy *c*-means, and level set, were compared in [18], the level set being a more accurate approach. In [19], a novel lazy snapping method was presented for detecting hot or cold regions in medical thermographic images to segment different diseases in the breast, foot, knees, lower back, and abdomen.

Next, we review related papers on thermographic breast image classification [1, 3, 4, 6, 20–30], which is the topic of our work. Two different kinds of neural network classifiers have been compared: a feedforward neural network and a radial basis function classifier [20]. Breast cancer analysis was performed using a series of statistical features extracted from the thermograms coupled with a fuzzy rule-based classification system for diagnosis [21]. Fractal analysis of breast thermal images was done to develop an algorithm [22]. The effectiveness of bispectral invariant features in the diagnostic classification of breast thermal images was evaluated, and a phase-only variant of these features was proposed. Classification was done using AdaBoost [4].

In another study [6], the diagnostic power of thermography in breast cancer was evaluated using 16 qualitative and explanatory variables and hill climbing classifiers. Araújo et al. proposed a three-stage feature extraction approach using Fisher's criterion and minimum distance classifiers (Euclidean distance) [3]. Rotational thermography techniques were evaluated, and texture features were extracted in the spatial domain and fed to a support vector machine (SVM) for automatic classification [23]. A system was presented based on 20 gray level co-occurrence matrices with feature extraction and classification by the *k*-nearest neighbors method [24]. An expert system was developed based on the measured temperature gradients (ΔT) in thermograms and classified them as normal, abnormal (ΔT > 2.5, <3), and potentially having breast cancer (ΔT ≥ 3) [25].

**Figure 1:**
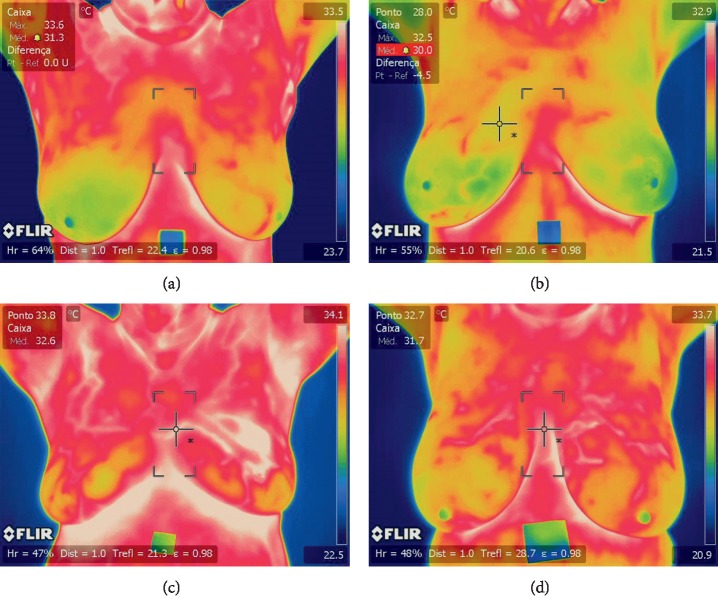
Input images. (a, b) Thermographic breast image without cancer. (c, d) Thermographic breast image with cancer.

A computer-aided detection (CAD) system was proposed with a segmentation approach based on both neutrosophic sets and the optimized fast fuzzy *c*-mean method [26]. Statistical, texture, and energy features were extracted and then classified by the SVM. Another method extracted statistical features and fed them to a nearest-neighbors classifier [27]. Hot spots and warm spots have also been detected in each view and region of interest, and features were extracted from them to feed SVMs and random forests [28]. A breast cancer detection algorithm was proposed based on texture feature extraction, a Markov random field, and a modified local binary pattern. Classification was done by a decision-level fusion algorithm by means of a hidden Markov model [29]. An asymmetry approach was proposed using the detection of any type of abnormalities (MC, masses, etc.) and bilateral subtraction [30]. Another method extracts 20 characteristics of the relationship of temperatures and classifies them by sequential minimal optimization [1]. Basically, our research is one of the next logical steps in progressing thermography in the breast cancer classification field by using convolutional neural networks and also presents a novel method that has not been used for the segmentation of breasts. CNNs have recently been used in several applications, including hand-written digit recognition, face detection, face recognition, and different medical applications [31–35]. Here, we focus on breast thermography images to identify cancer by CNN. We classify these data into normal (Figures 1(a) and 1(b)) and abnormal (Figures 1(c) and 1(d)); these images were evaluated and classified by two medical experts.

**Figure 2:**
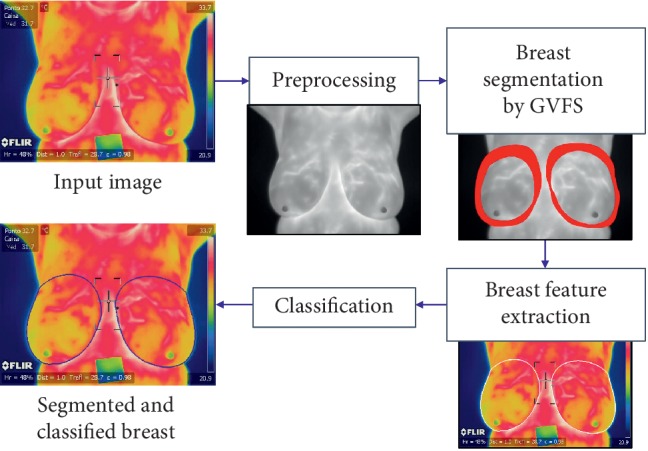
Proposed GFV segmentation method for breast cancer identification.

In this paper, we present an effective and efficient method to segment thermographic breast images and identify breast cancer for classification as normal or abnormal (without cancer or with cancer (Figure 2). The main contributions are the novel use of the combination curvature function *k* (cvt *k*) and gradient vector flow method (GVF) for breast segmentation, and for the analysis and classification of the segmented thermographic images, we proposed the use of a convolutional neural network (CNN); we also present the comparison of CNN classification results with tree random forest (TRF), multilayer perceptron (MLP), and Bayes network (BN).

The paper is organized as follows.  Section 2 describes the proposed segmentation and classification algorithms for breast thermographic images (Figure 2).  Section 3 presents the identification results obtained by applying different classification strategies in the most difficult validation scheme (2-fold cross validation).  Section 4 discusses the results and concludes the paper.

## 2. Materials and Methods

Figure 2 illustrates the stages of the proposed segmentation algorithm and classification techniques implemented. These are detailed in the following sections. The proposed system is accomplished in four stages: image preprocessing RGB and gray input, image denoising, and curvature function *k* (cvt *k*) for initial elliptical points for the GVF and classification; we then nest the breast image segmentation by gradient vector flow snake (GVF): following first by feature extraction (shape, colour, texture, and left and right breast relation) for feeding the three classification techniques TRF, MLP, and BN in comparison with the CNN; finally, we classify the segmented images as normal or abnormal with CNN using only the segmented regions of interest obtained by cvt *k* and GVF. The proposed technique is applicable to all breasts modifying the parameters of the cvt *k* and GVF.

### 2.1. Preprocessing for Initial Elliptical Points

The input RGB breast images are for feature extraction, and the input gray breast images are for preprocessing and segmentation (Figure 2, image provided by Silva et al. [11]). The input gray breast image is first denoised with a Gaussian filter (3 × 3). The perceptual linearity makes it more suitable for gradient vector flow snake implementation for the breast region segmentation, which is the following step.

**Figure 3:**
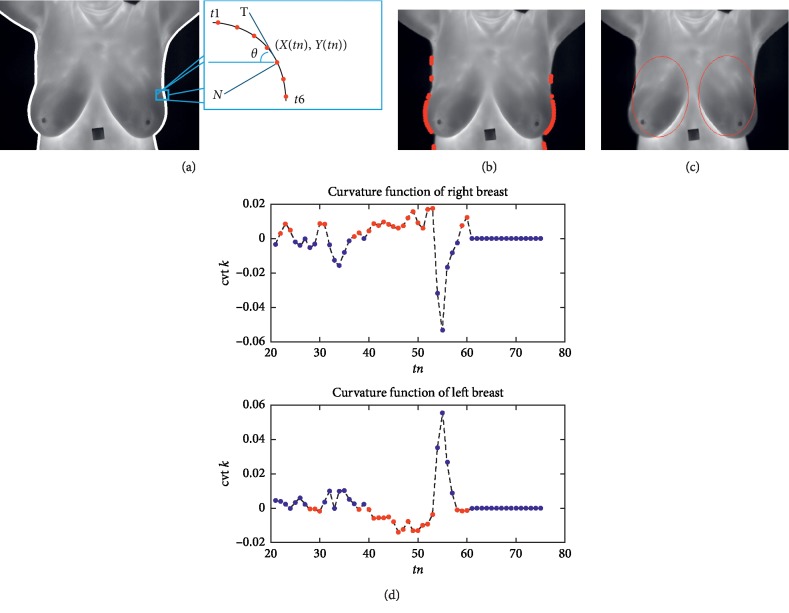
Initial elliptical points for GVF. (a) Left and right margins or lines. (b) Boundaries from curvature function. (c) Initial elliptical points obtained from cvt *k* for GVF. (d) Curvature function of right breast and left breast ([Supplementary-material supplementary-material-1] (MPEG, 169 KB) of supplementary materials).

We start from the observation that the breasts are anatomically elliptical. Boundary analysis used the initial snake for the GVF. Boundary analysis using the curvature function *k* identified the salient points on the curve. The input gray thermographic breast image filtered was first converted to binary using a predefined threshold of 0.25. Later, these were defined as left and right margins via canny edge detection (lines in Figure 3(a)). These two margins or lines represent two closed object boundaries via a sequence of points *C* = {(*xn*, *yn*)}, where *xn* = *x*(*tn*) and *yn* = *y*(*tn*). The curvature at a point on this planar curve (defined by the sequence of boundary points) is the rate of change of the angle with respect to arc length *k* = *dθ*/*ds*. Here, *s* is the arc length parameter. The curvature is a local geometric property of the curve.

The tangent vector *T* shown in Figure 3(a) is defined by $T={\left[\begin{array}{cc} \dot{x}\; & \dot{y} \end{array}\right]}^{T}$. The normal vector *N*, which is perpendicular to the tangent vector, is given by $N={\left[\begin{array}{cc} -\dot{y} & \dot{x} \end{array}\right]}^{T}$. The tangent of the angle *θ* at (*x*(*tn*), *y*(*tn*)) is given by $\tan\;\theta =dy/dx=\dot{x}/\dot{y}$. It can then be shown that the curvature *k* of the parametric curve can be written as $\text{cvt }k=\ddot{x}\dot{y}-\left({\dot{x}\ddot{y}/\left({\dot{x}}^{2}+{\dot{y}}^{2}\right)}^{3/2}\right)$.

Figure 3(b) shows the boundaries on which the positive and negative curvatures are labelled with different red markers in the right and left breasts. The two graphs shown in Figure 3(d) depict the curvature function cvt *k* for the right and left breasts. In the cvt *k* of the right breast, we emphasize the positive peaks. The negative peaks are emphasized in the cvt *k* of the left breast; peaks of interest are in red in both figures. Note that in the right breast, the cvt *k* is positive when the boundary line is concave. It is negative in the left breast when the boundary curve is convex. These features indicate the initial elliptical points of interest for the gradient vector flow snake. Thus, the curvatures of the boundary curves carry unique signatures that we utilized for right and left breast identification via the morphological contraction of these two elliptical objects defined in red (Figure 3(c)).

### 2.2. Breast Segmentation by Gradient Vector Flow Snakes

**Figure 4:**
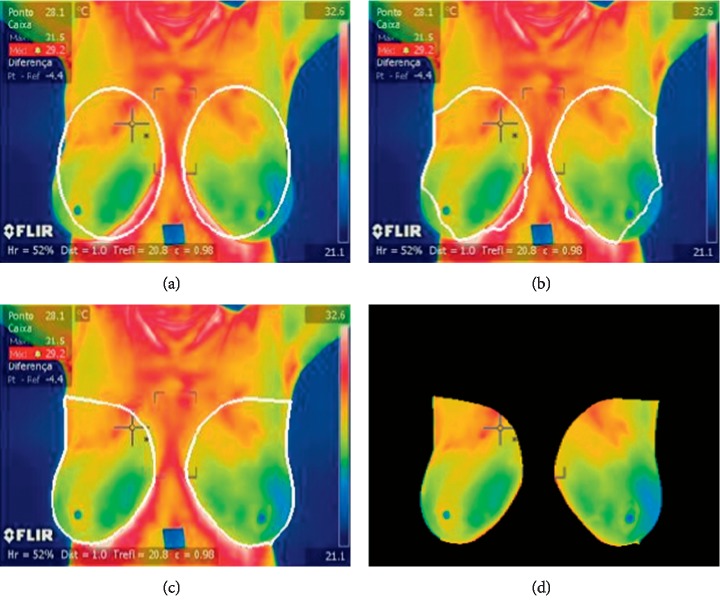
GVF segmentation of the breast region of interest. (a) Initial, (b) 200 iterations, (c) 5000 iterations, and (d) segmented CNN input image ([Supplementary-material supplementary-material-1] (MPEG, 489 KB) of supplementary materials).

Once an image is preprocessed, the list of initial points is then initialized for left and right breasts (Figure 4); we start from the observation that regions belonging to a breast should be elliptical and nearly similar. Thus, the list of initial points forms ellipses. These two ellipses can work like initial points for the GVF snakes may be applied. We followed the GVF method of our previous work, concerning automated pollen grain detection and classification from earlier microscopic prepared images [36].

Traditional snakes are curves (*v*(*s*) = [*x*(*s*), *y*(*s*)], *s* ∈ [0, 1]) defined within the domain of an image; it can move itself under the influence of internal forces coming from within the curve itself and external forces computed from the image data as first introduced by Kass et al. [37]. The GVF improves the capture range of the contours obtained by the binary image. Xu and Prince [38] proposed an improved snake to obtain better performance for image segmentation (Figure 4).

The formulation of a GVF is valid for gray images as well as binary images; however, we used gray images as seen in Figures 2 and 3. To compute GVFS, an edge-map function is first calculated using a Gaussian function. The initial values are based on *a priori* knowledge and several experiments for both breasts. *α* specifies the elasticity of the snake, and this controls the tension in the contour by combining with the first derivative term (*alpha* = 0.20). *β* specifies the rigidity in the contour by combining with the second derivative term (*beta* = 0.20). *γ* specifies the step size (*gamma* = 1.00). *κ* acts as the scaling factor for the energy term (*kappa* = 0.1). The *wEline* weighting factor is used for the intensity-based potential term (*wl* = 0.01). The *wEedge* weighting factor is for the edge-based potential term (*we* = 0.40). The *wEterm* weighing factor is for the termination potential term (*wt* = 0.01). The user then specifies the number of iterations for which the contour's position is to be computed with iterations of 5000. An edge-map function and an approximation of its gradient are then given. The GVFS is computed to guide the deformation of the snake at the boundary edges. Figure 4 shows the results of variations in breast segmentation and classification based on RGB thermographic breast images.

The segmented CNN input image is shown in Figure 4(d). This phase is designed to maximize recall and avoid false negatives of TRF, MLP, and BN. This is a critical factor in medical imaging. The objective of the following CNN phases (Section 2.4) is to maximize the precision and remove, or at least to identify, false candidates. The specific values of the a priori restrictions are quite flexible because they should avoid missing true positives. We first obtained the initial results, which are presented in the following section (Section 2.3), and compared the results of TRF, MLP, and BN with those of CNN.

### 2.3. Feature Extraction and Classification

This part aims to characterize the segmented breast with a feature vector that helps identify cancer in the thermographic breast images. These breast regions are then mapped on the R ∗ G ∗ B ∗ colour model image for feature extraction. The selected features can be grouped into four categories (Table 1).

#### 2.3.1. Shape Descriptors

The shape differences between left and right breast give clues for classification. First, the area (*A*) of the grain is determined by counting the number of pixels within the border, and the perimeter (*P*) is the length of the border. The regions *A* and *P* can be used as descriptors because of the differences in size between breasts; this is a medical parameter of interest. The roundness (*R*) is defined as the multiplication of 4*π* and *A* over *P*^*2*^. If *R* = *1*, then the object is circular. The compactness (*C*) is defined as the result of *A* over *P*. Each breast (left and right) gave eight terms: *A*_*l*_, *A*_*r*_, *P*_*l*_, *P*_*r*_, *R*_*l*_, *R*_*r*_, *C*_*l*_, and *C*_*r*_.

#### 2.3.2. First-Order Texture Descriptors

One way to discriminate between different textures is to compare R^*∗*^, G^*∗*^, and B^*∗*^ levels using first-order statistics. Red indicates high temperatures related to breast cancer. First-order statistics are calculated based on the probability of observing a particular pixel value at a randomly chosen location in the image. They depend only on individual pixel values and not on the interaction of neighboring pixel values. The average (*µ*) is the mean of the sum of all intensity values in the image. The median (*m*) represents the value of the central variable position in the dataset of sorted pixels. The variance (*σ*^2^) is a dispersion measure defined as the squared deviation of the variable with respect to its mean. Standard deviation (*σ*) is a measure of centralization or dispersion variable. Entropy (*S*) of the object in the image is a measure of content information. For both left and right breasts (*µ*_*l*_, *µ*_*r*_, *m*_*l*_, *m*_*r*_, *σ*_*l*_^2^, *σ*_*r*_^2^, *σ*_*l*_, *σ*_*r*_, *S*_*l*_, and *S*_*r*_), R^∗^, G^∗^, and B^∗^ levels gave 30 features.

#### 2.3.3. Second-Order Texture Descriptors

Haralick's gray-level co-occurrence matrices [39] have been used very successfully for biomedical image classification [40, 41]. Out of 14 features outlined, we first considered four texture features suitable for our experiment. We propose to use the co-occurrence matrix for the entire R^∗^G^∗^B^∗^ colour model. Contrast descriptor (CM) is a measure of local variation in the image. It is a high value when the region within the range of the window has high contrast. Correlation (*r*) of the texture measures the relationship between the different intensities of colours. Mathematically, the correlation increases when the variance is low, suggesting that the matrix elements are not far from the main diagonal. Energy (*e*) is the sum of the squared elements in the matrix of co-occurrence of gray level, also known as the uniformity or the second element of the angular momentum. Local homogeneity (HL) provides information on local regularity of the texture. The value of the local homogeneity is higher when the elements of the co-occurrence matrix are closer to the main diagonal. Both the left and right breasts (CM_*l*_, CM_*r*_, *r*_*l*_, *r*_*r*_, *e*_*l*_, *e*_*r*_, HL_*l*_, and HL_*r*_) were classified via the R^*∗*^, G^*∗*^, and B^*∗*^ levels to give 24 features.

#### 2.3.4. Relation Context Features

Relation breast context features are selected to capture the value of the relation between left and right breasts in the R^*∗*^, G^*∗*^, and B^*∗*^ levels considering the asymmetries between the breasts as an abnormality indicator. This yields values for relationships with respect to the left and right features: Euclidean distances, Bhattacharyya distance (BD), and absolute differences (*D*). The shape gave 12 features, the first-order texture gave 45 features, and the second-order texture gave 36 features. Thus, there were 93 relation context features.

#### 2.3.5. Classification

**Figure 5:**
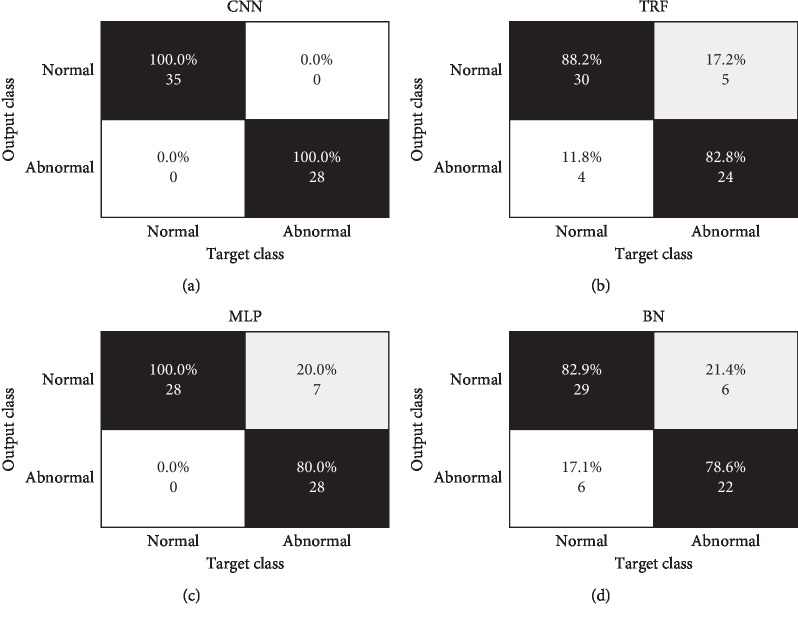
Confusion matrices of (a) CNN, (b) TRF, (c) MLP, and (d) BN.

The main aim of this work is to compare the proposed segmented image and the CNN classification with the characterization and classification of breasts using three classification techniques. In order to classify the segmented breast into normal or abnormal, identify cancer, and obtain final classification results, we explored the use of three different classification approaches implemented in Weka (Waikato Environment for Knowledge Analysis) [42, 43]: tree random forest (TRF) [44], multilayer perceptron (MLP), and Bayes network (BN). The experimental results obtained for these three classification techniques are compared with CNN classification (see results in Figure 5, Table 2, and Section 3).

### 2.4. CNN Classification

**Figure 6:**
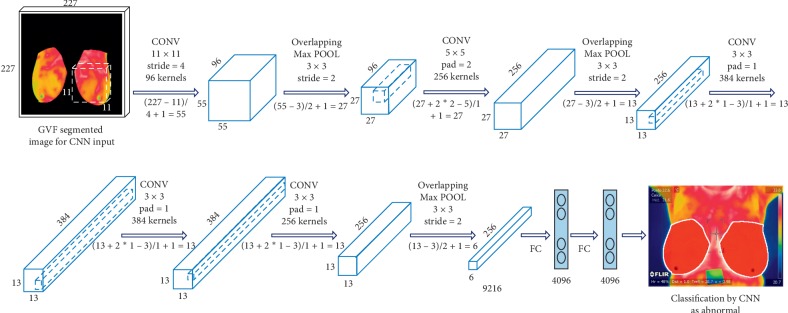
Convolutional neural network.

We here present an effective and efficient CNN classification system (Figure 6). Originally proposed by LeCun et al. [45], a CNN is a neural network model with three key architectural ideas: local receptive fields, weight sharing, and subsampling in the spatial domain. A CNN consists of three main types of layers designed to obtain the feature maps: spatial convolution layers (Cl), subsampling pooling layers (Sl), and fully connected layers (Fl)—l is a layer index.

We use a CNN based on previous studies designed to process two-dimensional (2D) images [46]. *C*l and *S*l layers are 2D layers, whereas the Fl layer and output are 1D layers. The motivation is that a CNN is advantageous for only breast thermography image in that it is hierarchical (with multiple layers for more compactness and efficiency) and invariance-redundant (for position, size, luminance, rotation, pose-angle, noise, and distortion).

We propose an efficient method to classify the segmented breast thermography image with a gradient vector flow to feed the CNN. We demonstrate that a classification method using the segmented breast to feed CNN is more robust and efficient than conventional state-of-the-art (SoA) methods using only classical features and classification techniques (Section 2.3.5). Our main contributions are as follows: the generation of segmented input images, capturing relevant breast information, and training and feeding the CNN for the comparison and evaluation of several classification strategies to confront the classification problem—TRF, MLP, and BN. For every thermography breast image, we generate a segmented image input to capture the semantics of the breast. These image are modified to 277 × 277 × 3 RGB images to feed the CNN (Figure 6).

## 3. Experimental Results

### 3.1. Dataset Description

**Figure 7:**
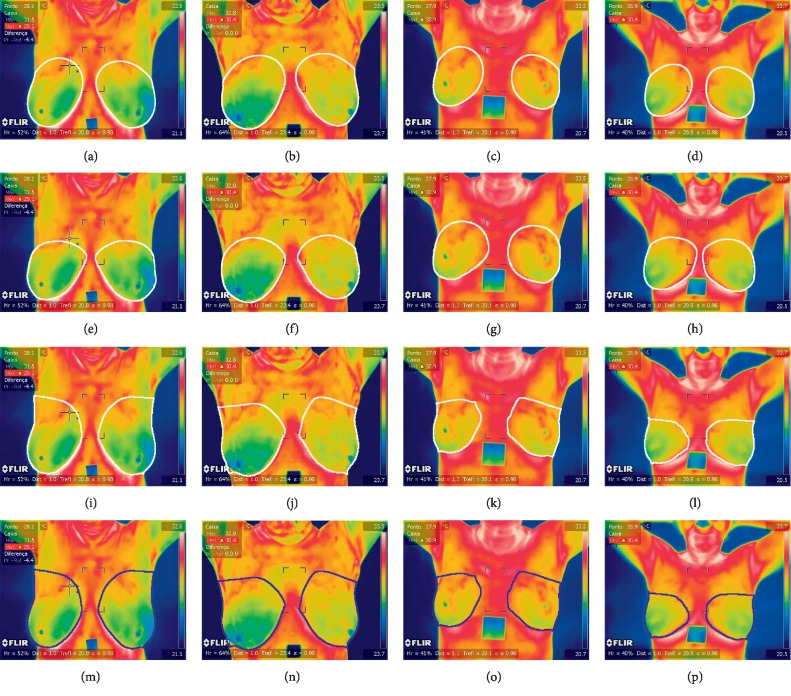
Thermographic breast image without cancer ([Supplementary-material supplementary-material-1] (MPEG, 489 KB) of supplementary materials). Ground truth from the first medical expert (a–d), ground truth from the second medical expert (e–h), segmentation (i–l), and classification results (m–p).

**Figure 8:**
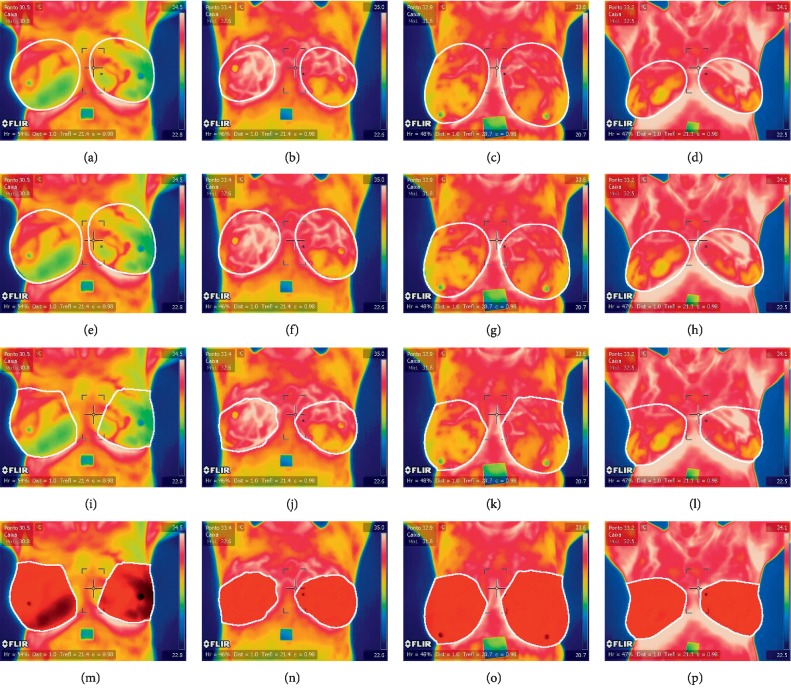
Thermographic breast image with cancer ([Supplementary-material supplementary-material-1], MPEG, 489 KB). Ground truth from the first medical expert (a–d), ground truth from the second medical expert (e–h), segmentation (i–l), and classification results (m–p).

The dataset includes 63 thermographic images (35 normal and 28 abnormal) in RGB colour format and in JPEG image format with a size of 680 × 480 × 3 as kindly provided by Silva et al. [11] and can be downloaded from [47]. There are 155 × 35 normal features and 155 × 28 abnormal features to test and train TRF, MLP, and BN obtained from 63 segmented CNN input images (35 normal and 28 abnormal). However, the proposed method can be easily adapted to different thermographic breast images.  Figure 1 shows example images of the dataset, and Figures 7 and 8 show the ground truth from two medical experts, segmentation and classification results of these images. The ground-truth data for the segmentation and classification of breast tissue and their degree of alteration with respect to different temperature levels were obtained from two oncologists via breast localization. We then present very similar segmentation results to those of medical experts according to the Zijdenbos Similarity Index with excellent result classification.

### 3.2. Quality Indicators of Thermographic Breast Cancer Image Classification

Several quality indicators have been obtained to quantitatively assess the breast classification results and the performance of the CNN, TRF, MLP, and BN techniques. We have divided them into final and external quality indicators, which evaluate the final segmentation results and are useful for external comparison with other works, and internal quality indicators, which are useful for evaluating the internal behavior of the proposed classification options.

For external indicators, let *P* be the number of normal breasts in the dataset, and let TP, FP, and FN be the number of true positives, false positives, and false negatives, respectively (Figure 5). We then define the following (see Table 2): sensitivity, recall, or true-positive rate: TPR = TP/P; precision or positive predictive value: PPV = TP/TP + FP; false discovery rate: FDR = FP/FP + TP; and the *F*1 score, overlap, or harmonic mean of TPR and PPV: *F*1 = 2 *∗* TP/2 *∗* TP + FP + FN, HM = 2 *∗* TPR *∗* PPV/TPR + PPV.

As the proposed algorithm will classify breast temperature regions of interest, which are then characterized and separated into normal and abnormal, we can further evaluate the classification performance of the four selected classification schemes via internal indicators. Let *N* be the number of abnormal breasts with cancer resulting from the application of the proposed method to the complete dataset, and let TN be the number of true negatives after classification (Figure 5). We can then define the following (see Table 2): specificity or true negative rate: SPC = TN/N; negative predictive value: NPV = TN/TN + FN; accuracy: ACC = TP + TN/TP + FP + TN + FN; fall-out or false-positive rate: FPR = FP/N; and the area under the receiver operating characteristic curve: AUC.

### 3.3. Quantitative and Qualitative Evaluation of Thermographic Breast Cancer Image Classification

There were 63 thermographic images (35 normal and 28 abnormal) for a total of 155 × 35 normal features and 155 × 28 abnormal features. The segmented breasts and the feature extraction phases for the described dataset include a collection of 155 total extracted features from 63 different breast images (35 normal breast and 28 expected abnormal breast). A 155-dimension feature vector extracted from shape, first-order texture, second-order texture, and relation context features characterized each segmented breast. As mentioned, three representative classification techniques were explored (BN, MLP, and TRF) using the toughest but most realistic classification experiment involving 2-fold cross validation schemes (*s* = 2) for training and testing. Figure 5 and Table 2 summarize the quantitative results of CNN, BN, MLP, and TRF. CNN achieved the best results in 2-fold cross validation where the dataset is divided into two equal parts: the first part is used for training and the second is used for testing. This was later switched: the second part was used for training and the first part for testing. This proves that these classifiers are reasonable for the classification of breast thermographic images and confirms the advantages of CNN over other state-of-the-art classifiers.

Qualitative results of breast classification are shown in Figures 7 and 8 with white (for ground truth and segmentation) and red and blue regions superimposed over correctly detected normal breast (TP, in blue in Figures 7(m)–7(p)) and correctly classified abnormal breast (TN, in red in Figures 8(m)–8(p)). These illustrate the good performance of the feature extraction and classification phases. The results show that the proposed classification method can successfully classify breast even in challenging environments.

### 3.4. Quantitative and Qualitative Evaluation of Nuclei Segmentation

Two medical experts defined a region around both breasts to define ground truth (histologically confirmed diagnosis) comparison (see Figures 7(a)–7(h)) and 8(a)–8(h)). Thus, good segmentation and precise breast cancer classification are both desired. To assess segmentation quality, we compared the region associated with a correctly identified breast and the corresponding region in the ground truth. The comparisons were quantified using the Zijdenbos Similarity Index, ZSI = 2 *∗* *|A*1 ∩ *A*2*|*/(*|A*1*| + |A*2*|*), where *A*1 and *A*2 refer to the compared regions and are both binary masks. A ZSI value greater than 0.75 indicates excellent agreement [48].

The ground truth includes segmentation data from two different experts referred to as GT1 and GT2. The two expert results comprise regions associated with correctly identified breast segmentation by GVF.  Table 3 summarizes the statistics for the ZSI obtained for every possible pair of experts (*A*1 and *A*2 comparison). The ZSI for the GVF compared with the GT1 had a mean of 0.8177 and a standard deviation of 0.0173; with GT2, the mean was 0.8229 and the standard deviation was 0.0094. This proves that the proposed segmentation approach gives similar results as those obtained manually by an expert.

### 3.5. Comparative Discussion

Publicly accessible datasets or evaluation scenarios that allow for a fair comparison among methods are lacking, and the code for reported methods is unavailable. Thus, we have chosen to present just our results on thermographic breast image classification.

One study [20] showed the feasibility of applying an ANN for the early detection and differentiation of abnormal patient states in health screening; their classifying systems are effective to the tune of more than 92% accuracy. Another study [21] concluded that the presented approach is indeed useful as an aid for the diagnosis of breast cancer and should prove even more powerful when coupled with another modality such as mammography; their approach provides a classification accuracy of about 80%. Yet another study [22] suggested that fractal analysis may potentially improve the reliability of thermography in breast tumor detection, with an accuracy of 90%. It has been shown that higher-order spectral features are capable of differentiating between different classes such as malignant, benign, and normal tissue in breast thermograms [4]; malignant cases are detected with 95% accuracy.

The framework of Bayesian networks provides a good model for analyzing thermographic breast images [6], obtaining an accuracy of 71.88%. A new feature extraction approach was presented for breast thermography classification [3]. This approach combines morphological, mathematical, and symbolic data analysis operators to discriminate different classes such as malignant, benign, and cyst tissue in breast thermograms; they reported a 85.7% of sensitivity and 86.5% of specificity to the malignant class. A pilot study [23] evaluated the potential of rotational thermography for automatic detection of breast abnormalities from the perspective of cold challenge; the accuracy of the classification system is found to be better than 83%. The goal of another work [24] was to compare the classification results of three different classifiers (SVM, *k*-NN, and Naive Bayes) and use GLCM features extracted from each thermography image; they obtained the accuracy ratio of 92.5%, which corresponded to true positive fraction of 78.6% at a false positive fraction of 0%. In another pilot study [25], digital infrared thermal imaging showed promising results and is thus well suited as a screening tool, obtaining a sensitivity of 97.6%, specificity of 99.17%, positive predictive value of 83.67%, and negative predictive value of 99.89%. Its use in combination with other laboratory and outcome assessment tools could lead to a significant improvement in the management of breast cancer.

Other results [12] indicate that thermography has the necessary sensitivity to effectively and inexpensively provide such an assessment; a sensitivity of 100% was claimed in their work (full train and full test). Another study [26] used several features (statistical, texture, and energy) with the SVM to detect normal and abnormal breast tissue; their system was achieving an excellent result of 100% using leave-one-out cross validation. Other results [27] showed that using simple texture descriptors in combination with a nearest-neighbors classifier can detect the early onset of breast tumors in women of any age, where abnormal breasts were identified with an accuracy of 94.44%. A potential breakthrough was indicated in thermographic screening for breast cancer [28]; they were able to achieve around 99% specificity while having 100% sensitivity. Other results [29] indicated that useful features of texture can be extracted with MRF models, LBPc, and LBPe and decision-level fusion-based classification using HMM on thermography images to achieve 87% accuracy. A CADx methodology dedicated to the creation of patient features combined the information of contralateral asymmetry and different views into single feature [30]; an area under the roc curve of 73.8% and 76.7% was achieved. The WEKA software SMO classifier obtained more expressive results regarding the diagnosis of breast abnormalities [1], achieving 93.42% accuracy, 94.73% sensitivity, and 92.10% specificity for the cancer class in a binary (cancer versus noncancer) analysis.

In light of these studies, we can confirm to some extent that our approach is valid based on our CNN classification results (TPR = 100% and PPV = 100% for a dataset containing 73 breast images, using 2-fold cross validation) in comparison with other similar “state-of-the-art” studies.

## 4. Conclusion

The main objective of this work is to make scientific contributions to a biomedical system for the acquisition of thermographic images of breasts via image processing. This provides a prediagnosis of breast cancer via GVF in combination with CNN. This paper proposes a novel method of initial selection of areas of interest in the chest through the analysis of the cvt *k* in both the right and left chest. The initial regions of interest of both breasts then feed the GVF technique to extract the characteristics for an accurate classification of the segmented regions. Finally, this determines the difference between the normal cases (without cancer) and abnormal cases (with cancer).

This work shows that a classification method that uses the combination of breast segmentation by GVF and applying CNN classification can be robust and efficient. Our main contributions include the novel segmentation via GVF of the region of interest of the thermographic image of the sinuses; segmentation of these input images to capture relevant information from the breasts to train and feed CNN, BN, MLP, and TRF with the segmented image or with feature extraction; the generation of a set of representative data with ground-truth data by specialist physicians to compare with our segmentation technique; and the evaluation of four classification strategies (CNN, BN, MLP, and TRF). We compared our data to the state of the art and observed that this approach gave results between 80% and 100% for TPR, SPC, and ACC. Thus, this approach improves outcomes and accuracy.

We demonstrated that a combination of GVF and CNN can detect breast cancer via the classification of thermographic images. The best results were obtained using CNN classifiers (100% TPR, SPC, and ACC). These results validate the novelty and quality of the proposed method. Future work will include a secondary framework to objectively compare our results.

## Figures and Tables

**Table 1:** Summary of descriptors.

*Shape*	
Area	*A*=*n*Pixels
Perimeter	$P=\sqrt{{\left(x_{i}-x_{i-1}\right)}^{2}+{\left(y_{i}-y_{i-1}\right)}^{2}}$
Roundness	*R*=4*π*(*A*/*P*^2^)
Compactness	*C*=*A*/*P*^2^

*First-order texture*	
Average	*μ*=1/*ij*∑_*i*,*j*_*p*(*i*, *j*)
Median	*m*=*L*+*I*((*N*/2) − *F*/*f*)
Variance	*σ* ^2^=1/*ij*∑_*i*,*j*_((*p*(*i*, *j*) − *μ*))
Standard deviation	$\sigma =\sqrt{1/ij\sum_{i,j}\left(p\left(i,j\right)-\mu \right)}$
Entropy	*S*=−∑_*i*,*j*_*p*(*i*, *j*)log *p*(*i*, *j*)

*Second-order texture*	
Contrast descriptor	CM=∑_*i*,*j*_|*i* − *j*|^2^*c*(*i*, *j*)
Correlation	*r*=∑_*i*,*j*_(*i* − *μ*_*ci*_)(*j* − *μ*_*cj*_)*c*(*i*, *j*)/*σ*_*ci*_*σ*_*cj*_
Energy	*e*=∑_*i*,*j*_*c*(*i*, *j*)^2^
Local homogeneity	HL=∑_*i*,*j*_*c*(*i*, *j*)/(1+|*i* − *j*|)

*Relation context*	
Euclidian distance	$\text{ED}=\sqrt{{\left(V_{r}-V_{l}\right)}^{2}}$
Bhattacharyya distance	$\text{BD}=\sqrt{\left(V_{r}\times V_{l}\right)}$
Difference	*D*=abs(*V*_*r*_ − *V*_*l*_)

**Table 2:** Quantitative classification results (%).

Technique	External quality indicators								
	TPR	PPV	FDR	*F*1 or HM	SPC	NPV	FPR	ACC	AUC
CNN	100	100	0	100	100	100	0	100	100
TRF	85.71	85.71	14.28	86.95	85.71	85.71	17.85	85.71	85.71
MLP	80	80	20	88.88	100	100	25	88.88	100
NV	82.85	82.85	17.14	82.85	78.57	78.57	21.42	80.95	78.57

ACC: accuracy; AUC: area under the receiver operating characteristic curve; *F*1: *F*1 score; FDR: false discovery rate; FPR: fall-out or false-positive rate; HM: harmonic mean; NPV: negative predictive value; PPV: precision or positive predictive value; SPC: specificity or true-negative rate; TPR: sensitivity, recall, or true-positive rate.

**Table 3:** ZSI statistics for segmentation results and ground truth.

	*A*2		
*A*1		GT1	GT2
	GVF	0.8177 ± 0.0173	0.8229 ± 0.0094

GT: ground truth; GVF: gradient vector flow segmentation result; ZSI: Zijdenbos Similarity Index.

## Data Availability

The data used to support the findings of this study are included within the supplementary material.

## Supplementary Materials

Supplementary Materials Figures 1–7: original images of Figures 1 to 7 in png format. MATLAB breast data: first run “AlexCNNbreast.m” code. Segmentation results of the breast images extracted from the gradient flow method (63 images in bmp format on folder “myImagesSEGMENTEDbreast” divided into normal and abnormal) to feed the convolutional neural network in Matlab2018a (“AlexCNNbreast.m” code to obtain the classification results and CNN models); the two CNN models from the 2-fold cross validation (“myNet_s1.mat” and “myNet_s2.mat”) that obtained 100% of TPR, SPC, and ACC; plotConfMat.m code to obtain the confusion matrices of CNN, TRF, MLP, and BN. Video results of Figures 3 and 4: VIDEO 1 of Figure 4 in mp4 format—this video describes the initial elliptical points for gradient vector flow using the curvature function *k* of right and left breasts; VIDEO 2 of Figure 4 in mp4 format—this video describes the gradient vector flow segmentation of the breast region of interest. WEKA breast data features: 155 × 63 classical features in Weka for TRF, MLP, and BN results. Run “BreastDatasetFeatures.arff” for obtaining the classification results.
