# Supplementary figures and images for: Breast Cancer Identification via Thermography Image Segmentation with a Gradient Vector Flow and a Convolutional Neural Network
Source: J Healthc Eng. 2019 Nov 3;2019:9807619. doi: 10.1155/2019/9807619 (PMC6935451; doi:10.1155/2019/9807619)

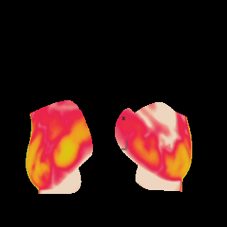

Supplement: Supplementary Materials — Figures 1–7: original images of Figures 1 to 7 in png format. MATLAB breast data: first run “AlexCNNbreast.m” code. Segmentation results of the breast images extracted from the gradient flow method (63 images in bmp format on folder “myImagesSEGMENTEDbreast” divided into normal and abnormal) to feed the convolutional neural network in Matlab2018a (“AlexCNNbreast.m” code to obtain the classification results and CNN models); the two CNN models from the 2-fold cross validation (“myNet_s1.mat” and “myNet_s2.mat”) that obtained 100% of TPR, SPC, and ACC; plotConfMat.m code to obtain the confusion matrices of CNN, TRF, MLP, and BN. Video results of Figures 3 and 4: VIDEO 1 of Figure 4 in mp4 format—this video describes the initial elliptical points for gradient vector flow using the curvature function k of right and left breasts; VIDEO 2 of Figure 4 in mp4 format—this video describes the gradient vector flow segmentation of the breast region of interest. WEKA breast data features: 155 × 63 classical features in Weka for TRF, MLP, and BN results. Run “BreastDatasetFeatures.arff” for obtaining the classification results. [file 9807619.f1.zip › 9807619_Addition_SupplementaryMaterials/MATLAB breast data/myImagesSEGMENTEDbreast/Abnormal/26.bmp]

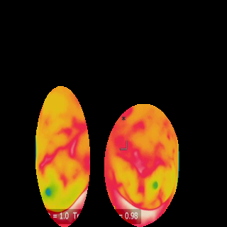

Supplement: Supplementary Materials — Figures 1–7: original images of Figures 1 to 7 in png format. MATLAB breast data: first run “AlexCNNbreast.m” code. Segmentation results of the breast images extracted from the gradient flow method (63 images in bmp format on folder “myImagesSEGMENTEDbreast” divided into normal and abnormal) to feed the convolutional neural network in Matlab2018a (“AlexCNNbreast.m” code to obtain the classification results and CNN models); the two CNN models from the 2-fold cross validation (“myNet_s1.mat” and “myNet_s2.mat”) that obtained 100% of TPR, SPC, and ACC; plotConfMat.m code to obtain the confusion matrices of CNN, TRF, MLP, and BN. Video results of Figures 3 and 4: VIDEO 1 of Figure 4 in mp4 format—this video describes the initial elliptical points for gradient vector flow using the curvature function k of right and left breasts; VIDEO 2 of Figure 4 in mp4 format—this video describes the gradient vector flow segmentation of the breast region of interest. WEKA breast data features: 155 × 63 classical features in Weka for TRF, MLP, and BN results. Run “BreastDatasetFeatures.arff” for obtaining the classification results. [file 9807619.f1.zip › 9807619_Addition_SupplementaryMaterials/MATLAB breast data/myImagesSEGMENTEDbreast/Abnormal/27.bmp]

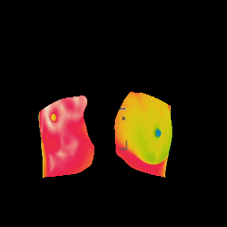

Supplement: Supplementary Materials — Figures 1–7: original images of Figures 1 to 7 in png format. MATLAB breast data: first run “AlexCNNbreast.m” code. Segmentation results of the breast images extracted from the gradient flow method (63 images in bmp format on folder “myImagesSEGMENTEDbreast” divided into normal and abnormal) to feed the convolutional neural network in Matlab2018a (“AlexCNNbreast.m” code to obtain the classification results and CNN models); the two CNN models from the 2-fold cross validation (“myNet_s1.mat” and “myNet_s2.mat”) that obtained 100% of TPR, SPC, and ACC; plotConfMat.m code to obtain the confusion matrices of CNN, TRF, MLP, and BN. Video results of Figures 3 and 4: VIDEO 1 of Figure 4 in mp4 format—this video describes the initial elliptical points for gradient vector flow using the curvature function k of right and left breasts; VIDEO 2 of Figure 4 in mp4 format—this video describes the gradient vector flow segmentation of the breast region of interest. WEKA breast data features: 155 × 63 classical features in Weka for TRF, MLP, and BN results. Run “BreastDatasetFeatures.arff” for obtaining the classification results. [file 9807619.f1.zip › 9807619_Addition_SupplementaryMaterials/MATLAB breast data/myImagesSEGMENTEDbreast/Abnormal/19.bmp]

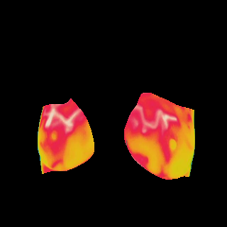

Supplement: Supplementary Materials — Figures 1–7: original images of Figures 1 to 7 in png format. MATLAB breast data: first run “AlexCNNbreast.m” code. Segmentation results of the breast images extracted from the gradient flow method (63 images in bmp format on folder “myImagesSEGMENTEDbreast” divided into normal and abnormal) to feed the convolutional neural network in Matlab2018a (“AlexCNNbreast.m” code to obtain the classification results and CNN models); the two CNN models from the 2-fold cross validation (“myNet_s1.mat” and “myNet_s2.mat”) that obtained 100% of TPR, SPC, and ACC; plotConfMat.m code to obtain the confusion matrices of CNN, TRF, MLP, and BN. Video results of Figures 3 and 4: VIDEO 1 of Figure 4 in mp4 format—this video describes the initial elliptical points for gradient vector flow using the curvature function k of right and left breasts; VIDEO 2 of Figure 4 in mp4 format—this video describes the gradient vector flow segmentation of the breast region of interest. WEKA breast data features: 155 × 63 classical features in Weka for TRF, MLP, and BN results. Run “BreastDatasetFeatures.arff” for obtaining the classification results. [file 9807619.f1.zip › 9807619_Addition_SupplementaryMaterials/MATLAB breast data/myImagesSEGMENTEDbreast/Abnormal/25.bmp]

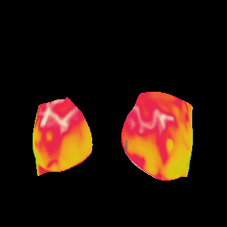

Supplement: Supplementary Materials — Figures 1–7: original images of Figures 1 to 7 in png format. MATLAB breast data: first run “AlexCNNbreast.m” code. Segmentation results of the breast images extracted from the gradient flow method (63 images in bmp format on folder “myImagesSEGMENTEDbreast” divided into normal and abnormal) to feed the convolutional neural network in Matlab2018a (“AlexCNNbreast.m” code to obtain the classification results and CNN models); the two CNN models from the 2-fold cross validation (“myNet_s1.mat” and “myNet_s2.mat”) that obtained 100% of TPR, SPC, and ACC; plotConfMat.m code to obtain the confusion matrices of CNN, TRF, MLP, and BN. Video results of Figures 3 and 4: VIDEO 1 of Figure 4 in mp4 format—this video describes the initial elliptical points for gradient vector flow using the curvature function k of right and left breasts; VIDEO 2 of Figure 4 in mp4 format—this video describes the gradient vector flow segmentation of the breast region of interest. WEKA breast data features: 155 × 63 classical features in Weka for TRF, MLP, and BN results. Run “BreastDatasetFeatures.arff” for obtaining the classification results. [file 9807619.f1.zip › 9807619_Addition_SupplementaryMaterials/MATLAB breast data/myImagesSEGMENTEDbreast/Abnormal/24.bmp]

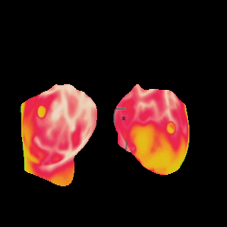

Supplement: Supplementary Materials — Figures 1–7: original images of Figures 1 to 7 in png format. MATLAB breast data: first run “AlexCNNbreast.m” code. Segmentation results of the breast images extracted from the gradient flow method (63 images in bmp format on folder “myImagesSEGMENTEDbreast” divided into normal and abnormal) to feed the convolutional neural network in Matlab2018a (“AlexCNNbreast.m” code to obtain the classification results and CNN models); the two CNN models from the 2-fold cross validation (“myNet_s1.mat” and “myNet_s2.mat”) that obtained 100% of TPR, SPC, and ACC; plotConfMat.m code to obtain the confusion matrices of CNN, TRF, MLP, and BN. Video results of Figures 3 and 4: VIDEO 1 of Figure 4 in mp4 format—this video describes the initial elliptical points for gradient vector flow using the curvature function k of right and left breasts; VIDEO 2 of Figure 4 in mp4 format—this video describes the gradient vector flow segmentation of the breast region of interest. WEKA breast data features: 155 × 63 classical features in Weka for TRF, MLP, and BN results. Run “BreastDatasetFeatures.arff” for obtaining the classification results. [file 9807619.f1.zip › 9807619_Addition_SupplementaryMaterials/MATLAB breast data/myImagesSEGMENTEDbreast/Abnormal/18.bmp]

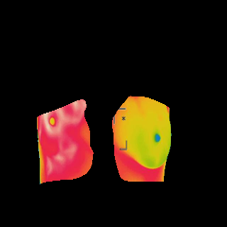

Supplement: Supplementary Materials — Figures 1–7: original images of Figures 1 to 7 in png format. MATLAB breast data: first run “AlexCNNbreast.m” code. Segmentation results of the breast images extracted from the gradient flow method (63 images in bmp format on folder “myImagesSEGMENTEDbreast” divided into normal and abnormal) to feed the convolutional neural network in Matlab2018a (“AlexCNNbreast.m” code to obtain the classification results and CNN models); the two CNN models from the 2-fold cross validation (“myNet_s1.mat” and “myNet_s2.mat”) that obtained 100% of TPR, SPC, and ACC; plotConfMat.m code to obtain the confusion matrices of CNN, TRF, MLP, and BN. Video results of Figures 3 and 4: VIDEO 1 of Figure 4 in mp4 format—this video describes the initial elliptical points for gradient vector flow using the curvature function k of right and left breasts; VIDEO 2 of Figure 4 in mp4 format—this video describes the gradient vector flow segmentation of the breast region of interest. WEKA breast data features: 155 × 63 classical features in Weka for TRF, MLP, and BN results. Run “BreastDatasetFeatures.arff” for obtaining the classification results. [file 9807619.f1.zip › 9807619_Addition_SupplementaryMaterials/MATLAB breast data/myImagesSEGMENTEDbreast/Abnormal/20.bmp]

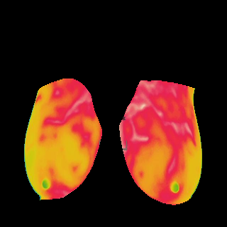

Supplement: Supplementary Materials — Figures 1–7: original images of Figures 1 to 7 in png format. MATLAB breast data: first run “AlexCNNbreast.m” code. Segmentation results of the breast images extracted from the gradient flow method (63 images in bmp format on folder “myImagesSEGMENTEDbreast” divided into normal and abnormal) to feed the convolutional neural network in Matlab2018a (“AlexCNNbreast.m” code to obtain the classification results and CNN models); the two CNN models from the 2-fold cross validation (“myNet_s1.mat” and “myNet_s2.mat”) that obtained 100% of TPR, SPC, and ACC; plotConfMat.m code to obtain the confusion matrices of CNN, TRF, MLP, and BN. Video results of Figures 3 and 4: VIDEO 1 of Figure 4 in mp4 format—this video describes the initial elliptical points for gradient vector flow using the curvature function k of right and left breasts; VIDEO 2 of Figure 4 in mp4 format—this video describes the gradient vector flow segmentation of the breast region of interest. WEKA breast data features: 155 × 63 classical features in Weka for TRF, MLP, and BN results. Run “BreastDatasetFeatures.arff” for obtaining the classification results. [file 9807619.f1.zip › 9807619_Addition_SupplementaryMaterials/MATLAB breast data/myImagesSEGMENTEDbreast/Abnormal/21.bmp]

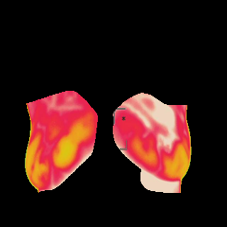

Supplement: Supplementary Materials — Figures 1–7: original images of Figures 1 to 7 in png format. MATLAB breast data: first run “AlexCNNbreast.m” code. Segmentation results of the breast images extracted from the gradient flow method (63 images in bmp format on folder “myImagesSEGMENTEDbreast” divided into normal and abnormal) to feed the convolutional neural network in Matlab2018a (“AlexCNNbreast.m” code to obtain the classification results and CNN models); the two CNN models from the 2-fold cross validation (“myNet_s1.mat” and “myNet_s2.mat”) that obtained 100% of TPR, SPC, and ACC; plotConfMat.m code to obtain the confusion matrices of CNN, TRF, MLP, and BN. Video results of Figures 3 and 4: VIDEO 1 of Figure 4 in mp4 format—this video describes the initial elliptical points for gradient vector flow using the curvature function k of right and left breasts; VIDEO 2 of Figure 4 in mp4 format—this video describes the gradient vector flow segmentation of the breast region of interest. WEKA breast data features: 155 × 63 classical features in Weka for TRF, MLP, and BN results. Run “BreastDatasetFeatures.arff” for obtaining the classification results. [file 9807619.f1.zip › 9807619_Addition_SupplementaryMaterials/MATLAB breast data/myImagesSEGMENTEDbreast/Abnormal/23.bmp]

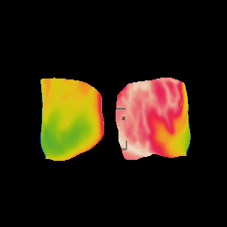

Supplement: Supplementary Materials — Figures 1–7: original images of Figures 1 to 7 in png format. MATLAB breast data: first run “AlexCNNbreast.m” code. Segmentation results of the breast images extracted from the gradient flow method (63 images in bmp format on folder “myImagesSEGMENTEDbreast” divided into normal and abnormal) to feed the convolutional neural network in Matlab2018a (“AlexCNNbreast.m” code to obtain the classification results and CNN models); the two CNN models from the 2-fold cross validation (“myNet_s1.mat” and “myNet_s2.mat”) that obtained 100% of TPR, SPC, and ACC; plotConfMat.m code to obtain the confusion matrices of CNN, TRF, MLP, and BN. Video results of Figures 3 and 4: VIDEO 1 of Figure 4 in mp4 format—this video describes the initial elliptical points for gradient vector flow using the curvature function k of right and left breasts; VIDEO 2 of Figure 4 in mp4 format—this video describes the gradient vector flow segmentation of the breast region of interest. WEKA breast data features: 155 × 63 classical features in Weka for TRF, MLP, and BN results. Run “BreastDatasetFeatures.arff” for obtaining the classification results. [file 9807619.f1.zip › 9807619_Addition_SupplementaryMaterials/MATLAB breast data/myImagesSEGMENTEDbreast/Abnormal/22.bmp]

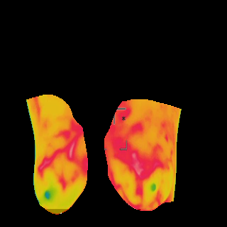

Supplement: Supplementary Materials — Figures 1–7: original images of Figures 1 to 7 in png format. MATLAB breast data: first run “AlexCNNbreast.m” code. Segmentation results of the breast images extracted from the gradient flow method (63 images in bmp format on folder “myImagesSEGMENTEDbreast” divided into normal and abnormal) to feed the convolutional neural network in Matlab2018a (“AlexCNNbreast.m” code to obtain the classification results and CNN models); the two CNN models from the 2-fold cross validation (“myNet_s1.mat” and “myNet_s2.mat”) that obtained 100% of TPR, SPC, and ACC; plotConfMat.m code to obtain the confusion matrices of CNN, TRF, MLP, and BN. Video results of Figures 3 and 4: VIDEO 1 of Figure 4 in mp4 format—this video describes the initial elliptical points for gradient vector flow using the curvature function k of right and left breasts; VIDEO 2 of Figure 4 in mp4 format—this video describes the gradient vector flow segmentation of the breast region of interest. WEKA breast data features: 155 × 63 classical features in Weka for TRF, MLP, and BN results. Run “BreastDatasetFeatures.arff” for obtaining the classification results. [file 9807619.f1.zip › 9807619_Addition_SupplementaryMaterials/MATLAB breast data/myImagesSEGMENTEDbreast/Abnormal/3.bmp]

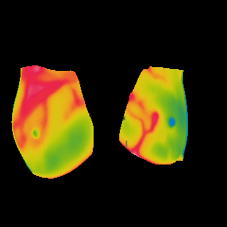

Supplement: Supplementary Materials — Figures 1–7: original images of Figures 1 to 7 in png format. MATLAB breast data: first run “AlexCNNbreast.m” code. Segmentation results of the breast images extracted from the gradient flow method (63 images in bmp format on folder “myImagesSEGMENTEDbreast” divided into normal and abnormal) to feed the convolutional neural network in Matlab2018a (“AlexCNNbreast.m” code to obtain the classification results and CNN models); the two CNN models from the 2-fold cross validation (“myNet_s1.mat” and “myNet_s2.mat”) that obtained 100% of TPR, SPC, and ACC; plotConfMat.m code to obtain the confusion matrices of CNN, TRF, MLP, and BN. Video results of Figures 3 and 4: VIDEO 1 of Figure 4 in mp4 format—this video describes the initial elliptical points for gradient vector flow using the curvature function k of right and left breasts; VIDEO 2 of Figure 4 in mp4 format—this video describes the gradient vector flow segmentation of the breast region of interest. WEKA breast data features: 155 × 63 classical features in Weka for TRF, MLP, and BN results. Run “BreastDatasetFeatures.arff” for obtaining the classification results. [file 9807619.f1.zip › 9807619_Addition_SupplementaryMaterials/MATLAB breast data/myImagesSEGMENTEDbreast/Abnormal/2.bmp]

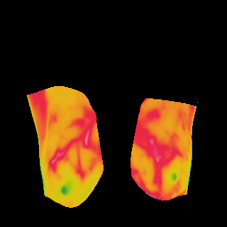

Supplement: Supplementary Materials — Figures 1–7: original images of Figures 1 to 7 in png format. MATLAB breast data: first run “AlexCNNbreast.m” code. Segmentation results of the breast images extracted from the gradient flow method (63 images in bmp format on folder “myImagesSEGMENTEDbreast” divided into normal and abnormal) to feed the convolutional neural network in Matlab2018a (“AlexCNNbreast.m” code to obtain the classification results and CNN models); the two CNN models from the 2-fold cross validation (“myNet_s1.mat” and “myNet_s2.mat”) that obtained 100% of TPR, SPC, and ACC; plotConfMat.m code to obtain the confusion matrices of CNN, TRF, MLP, and BN. Video results of Figures 3 and 4: VIDEO 1 of Figure 4 in mp4 format—this video describes the initial elliptical points for gradient vector flow using the curvature function k of right and left breasts; VIDEO 2 of Figure 4 in mp4 format—this video describes the gradient vector flow segmentation of the breast region of interest. WEKA breast data features: 155 × 63 classical features in Weka for TRF, MLP, and BN results. Run “BreastDatasetFeatures.arff” for obtaining the classification results. [file 9807619.f1.zip › 9807619_Addition_SupplementaryMaterials/MATLAB breast data/myImagesSEGMENTEDbreast/Abnormal/1.bmp]

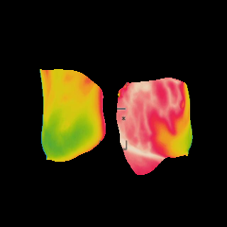

Supplement: Supplementary Materials — Figures 1–7: original images of Figures 1 to 7 in png format. MATLAB breast data: first run “AlexCNNbreast.m” code. Segmentation results of the breast images extracted from the gradient flow method (63 images in bmp format on folder “myImagesSEGMENTEDbreast” divided into normal and abnormal) to feed the convolutional neural network in Matlab2018a (“AlexCNNbreast.m” code to obtain the classification results and CNN models); the two CNN models from the 2-fold cross validation (“myNet_s1.mat” and “myNet_s2.mat”) that obtained 100% of TPR, SPC, and ACC; plotConfMat.m code to obtain the confusion matrices of CNN, TRF, MLP, and BN. Video results of Figures 3 and 4: VIDEO 1 of Figure 4 in mp4 format—this video describes the initial elliptical points for gradient vector flow using the curvature function k of right and left breasts; VIDEO 2 of Figure 4 in mp4 format—this video describes the gradient vector flow segmentation of the breast region of interest. WEKA breast data features: 155 × 63 classical features in Weka for TRF, MLP, and BN results. Run “BreastDatasetFeatures.arff” for obtaining the classification results. [file 9807619.f1.zip › 9807619_Addition_SupplementaryMaterials/MATLAB breast data/myImagesSEGMENTEDbreast/Abnormal/5.bmp]

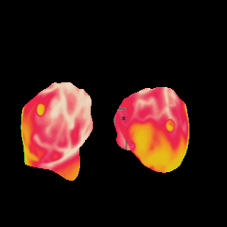

Supplement: Supplementary Materials — Figures 1–7: original images of Figures 1 to 7 in png format. MATLAB breast data: first run “AlexCNNbreast.m” code. Segmentation results of the breast images extracted from the gradient flow method (63 images in bmp format on folder “myImagesSEGMENTEDbreast” divided into normal and abnormal) to feed the convolutional neural network in Matlab2018a (“AlexCNNbreast.m” code to obtain the classification results and CNN models); the two CNN models from the 2-fold cross validation (“myNet_s1.mat” and “myNet_s2.mat”) that obtained 100% of TPR, SPC, and ACC; plotConfMat.m code to obtain the confusion matrices of CNN, TRF, MLP, and BN. Video results of Figures 3 and 4: VIDEO 1 of Figure 4 in mp4 format—this video describes the initial elliptical points for gradient vector flow using the curvature function k of right and left breasts; VIDEO 2 of Figure 4 in mp4 format—this video describes the gradient vector flow segmentation of the breast region of interest. WEKA breast data features: 155 × 63 classical features in Weka for TRF, MLP, and BN results. Run “BreastDatasetFeatures.arff” for obtaining the classification results. [file 9807619.f1.zip › 9807619_Addition_SupplementaryMaterials/MATLAB breast data/myImagesSEGMENTEDbreast/Abnormal/4.bmp]

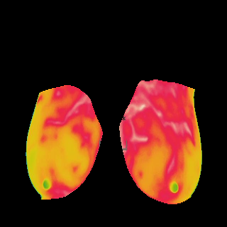

Supplement: Supplementary Materials — Figures 1–7: original images of Figures 1 to 7 in png format. MATLAB breast data: first run “AlexCNNbreast.m” code. Segmentation results of the breast images extracted from the gradient flow method (63 images in bmp format on folder “myImagesSEGMENTEDbreast” divided into normal and abnormal) to feed the convolutional neural network in Matlab2018a (“AlexCNNbreast.m” code to obtain the classification results and CNN models); the two CNN models from the 2-fold cross validation (“myNet_s1.mat” and “myNet_s2.mat”) that obtained 100% of TPR, SPC, and ACC; plotConfMat.m code to obtain the confusion matrices of CNN, TRF, MLP, and BN. Video results of Figures 3 and 4: VIDEO 1 of Figure 4 in mp4 format—this video describes the initial elliptical points for gradient vector flow using the curvature function k of right and left breasts; VIDEO 2 of Figure 4 in mp4 format—this video describes the gradient vector flow segmentation of the breast region of interest. WEKA breast data features: 155 × 63 classical features in Weka for TRF, MLP, and BN results. Run “BreastDatasetFeatures.arff” for obtaining the classification results. [file 9807619.f1.zip › 9807619_Addition_SupplementaryMaterials/MATLAB breast data/myImagesSEGMENTEDbreast/Abnormal/6.bmp]

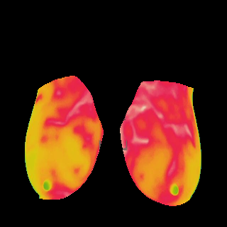

Supplement: Supplementary Materials — Figures 1–7: original images of Figures 1 to 7 in png format. MATLAB breast data: first run “AlexCNNbreast.m” code. Segmentation results of the breast images extracted from the gradient flow method (63 images in bmp format on folder “myImagesSEGMENTEDbreast” divided into normal and abnormal) to feed the convolutional neural network in Matlab2018a (“AlexCNNbreast.m” code to obtain the classification results and CNN models); the two CNN models from the 2-fold cross validation (“myNet_s1.mat” and “myNet_s2.mat”) that obtained 100% of TPR, SPC, and ACC; plotConfMat.m code to obtain the confusion matrices of CNN, TRF, MLP, and BN. Video results of Figures 3 and 4: VIDEO 1 of Figure 4 in mp4 format—this video describes the initial elliptical points for gradient vector flow using the curvature function k of right and left breasts; VIDEO 2 of Figure 4 in mp4 format—this video describes the gradient vector flow segmentation of the breast region of interest. WEKA breast data features: 155 × 63 classical features in Weka for TRF, MLP, and BN results. Run “BreastDatasetFeatures.arff” for obtaining the classification results. [file 9807619.f1.zip › 9807619_Addition_SupplementaryMaterials/MATLAB breast data/myImagesSEGMENTEDbreast/Abnormal/7.bmp]

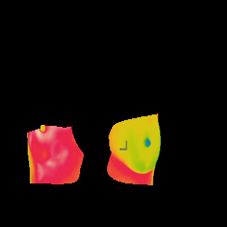

Supplement: Supplementary Materials — Figures 1–7: original images of Figures 1 to 7 in png format. MATLAB breast data: first run “AlexCNNbreast.m” code. Segmentation results of the breast images extracted from the gradient flow method (63 images in bmp format on folder “myImagesSEGMENTEDbreast” divided into normal and abnormal) to feed the convolutional neural network in Matlab2018a (“AlexCNNbreast.m” code to obtain the classification results and CNN models); the two CNN models from the 2-fold cross validation (“myNet_s1.mat” and “myNet_s2.mat”) that obtained 100% of TPR, SPC, and ACC; plotConfMat.m code to obtain the confusion matrices of CNN, TRF, MLP, and BN. Video results of Figures 3 and 4: VIDEO 1 of Figure 4 in mp4 format—this video describes the initial elliptical points for gradient vector flow using the curvature function k of right and left breasts; VIDEO 2 of Figure 4 in mp4 format—this video describes the gradient vector flow segmentation of the breast region of interest. WEKA breast data features: 155 × 63 classical features in Weka for TRF, MLP, and BN results. Run “BreastDatasetFeatures.arff” for obtaining the classification results. [file 9807619.f1.zip › 9807619_Addition_SupplementaryMaterials/MATLAB breast data/myImagesSEGMENTEDbreast/Abnormal/9.bmp]

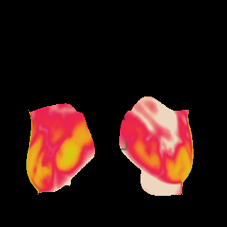

Supplement: Supplementary Materials — Figures 1–7: original images of Figures 1 to 7 in png format. MATLAB breast data: first run “AlexCNNbreast.m” code. Segmentation results of the breast images extracted from the gradient flow method (63 images in bmp format on folder “myImagesSEGMENTEDbreast” divided into normal and abnormal) to feed the convolutional neural network in Matlab2018a (“AlexCNNbreast.m” code to obtain the classification results and CNN models); the two CNN models from the 2-fold cross validation (“myNet_s1.mat” and “myNet_s2.mat”) that obtained 100% of TPR, SPC, and ACC; plotConfMat.m code to obtain the confusion matrices of CNN, TRF, MLP, and BN. Video results of Figures 3 and 4: VIDEO 1 of Figure 4 in mp4 format—this video describes the initial elliptical points for gradient vector flow using the curvature function k of right and left breasts; VIDEO 2 of Figure 4 in mp4 format—this video describes the gradient vector flow segmentation of the breast region of interest. WEKA breast data features: 155 × 63 classical features in Weka for TRF, MLP, and BN results. Run “BreastDatasetFeatures.arff” for obtaining the classification results. [file 9807619.f1.zip › 9807619_Addition_SupplementaryMaterials/MATLAB breast data/myImagesSEGMENTEDbreast/Abnormal/8.bmp]

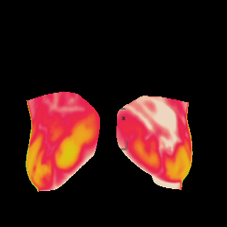

Supplement: Supplementary Materials — Figures 1–7: original images of Figures 1 to 7 in png format. MATLAB breast data: first run “AlexCNNbreast.m” code. Segmentation results of the breast images extracted from the gradient flow method (63 images in bmp format on folder “myImagesSEGMENTEDbreast” divided into normal and abnormal) to feed the convolutional neural network in Matlab2018a (“AlexCNNbreast.m” code to obtain the classification results and CNN models); the two CNN models from the 2-fold cross validation (“myNet_s1.mat” and “myNet_s2.mat”) that obtained 100% of TPR, SPC, and ACC; plotConfMat.m code to obtain the confusion matrices of CNN, TRF, MLP, and BN. Video results of Figures 3 and 4: VIDEO 1 of Figure 4 in mp4 format—this video describes the initial elliptical points for gradient vector flow using the curvature function k of right and left breasts; VIDEO 2 of Figure 4 in mp4 format—this video describes the gradient vector flow segmentation of the breast region of interest. WEKA breast data features: 155 × 63 classical features in Weka for TRF, MLP, and BN results. Run “BreastDatasetFeatures.arff” for obtaining the classification results. [file 9807619.f1.zip › 9807619_Addition_SupplementaryMaterials/MATLAB breast data/myImagesSEGMENTEDbreast/Abnormal/13.bmp]

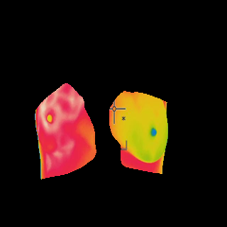

Supplement: Supplementary Materials — Figures 1–7: original images of Figures 1 to 7 in png format. MATLAB breast data: first run “AlexCNNbreast.m” code. Segmentation results of the breast images extracted from the gradient flow method (63 images in bmp format on folder “myImagesSEGMENTEDbreast” divided into normal and abnormal) to feed the convolutional neural network in Matlab2018a (“AlexCNNbreast.m” code to obtain the classification results and CNN models); the two CNN models from the 2-fold cross validation (“myNet_s1.mat” and “myNet_s2.mat”) that obtained 100% of TPR, SPC, and ACC; plotConfMat.m code to obtain the confusion matrices of CNN, TRF, MLP, and BN. Video results of Figures 3 and 4: VIDEO 1 of Figure 4 in mp4 format—this video describes the initial elliptical points for gradient vector flow using the curvature function k of right and left breasts; VIDEO 2 of Figure 4 in mp4 format—this video describes the gradient vector flow segmentation of the breast region of interest. WEKA breast data features: 155 × 63 classical features in Weka for TRF, MLP, and BN results. Run “BreastDatasetFeatures.arff” for obtaining the classification results. [file 9807619.f1.zip › 9807619_Addition_SupplementaryMaterials/MATLAB breast data/myImagesSEGMENTEDbreast/Abnormal/12.bmp]

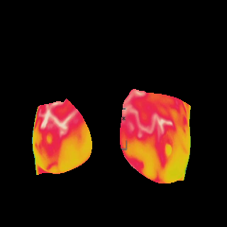

Supplement: Supplementary Materials — Figures 1–7: original images of Figures 1 to 7 in png format. MATLAB breast data: first run “AlexCNNbreast.m” code. Segmentation results of the breast images extracted from the gradient flow method (63 images in bmp format on folder “myImagesSEGMENTEDbreast” divided into normal and abnormal) to feed the convolutional neural network in Matlab2018a (“AlexCNNbreast.m” code to obtain the classification results and CNN models); the two CNN models from the 2-fold cross validation (“myNet_s1.mat” and “myNet_s2.mat”) that obtained 100% of TPR, SPC, and ACC; plotConfMat.m code to obtain the confusion matrices of CNN, TRF, MLP, and BN. Video results of Figures 3 and 4: VIDEO 1 of Figure 4 in mp4 format—this video describes the initial elliptical points for gradient vector flow using the curvature function k of right and left breasts; VIDEO 2 of Figure 4 in mp4 format—this video describes the gradient vector flow segmentation of the breast region of interest. WEKA breast data features: 155 × 63 classical features in Weka for TRF, MLP, and BN results. Run “BreastDatasetFeatures.arff” for obtaining the classification results. [file 9807619.f1.zip › 9807619_Addition_SupplementaryMaterials/MATLAB breast data/myImagesSEGMENTEDbreast/Abnormal/10.bmp]

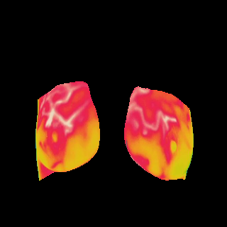

Supplement: Supplementary Materials — Figures 1–7: original images of Figures 1 to 7 in png format. MATLAB breast data: first run “AlexCNNbreast.m” code. Segmentation results of the breast images extracted from the gradient flow method (63 images in bmp format on folder “myImagesSEGMENTEDbreast” divided into normal and abnormal) to feed the convolutional neural network in Matlab2018a (“AlexCNNbreast.m” code to obtain the classification results and CNN models); the two CNN models from the 2-fold cross validation (“myNet_s1.mat” and “myNet_s2.mat”) that obtained 100% of TPR, SPC, and ACC; plotConfMat.m code to obtain the confusion matrices of CNN, TRF, MLP, and BN. Video results of Figures 3 and 4: VIDEO 1 of Figure 4 in mp4 format—this video describes the initial elliptical points for gradient vector flow using the curvature function k of right and left breasts; VIDEO 2 of Figure 4 in mp4 format—this video describes the gradient vector flow segmentation of the breast region of interest. WEKA breast data features: 155 × 63 classical features in Weka for TRF, MLP, and BN results. Run “BreastDatasetFeatures.arff” for obtaining the classification results. [file 9807619.f1.zip › 9807619_Addition_SupplementaryMaterials/MATLAB breast data/myImagesSEGMENTEDbreast/Abnormal/11.bmp]

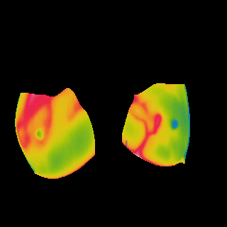

Supplement: Supplementary Materials — Figures 1–7: original images of Figures 1 to 7 in png format. MATLAB breast data: first run “AlexCNNbreast.m” code. Segmentation results of the breast images extracted from the gradient flow method (63 images in bmp format on folder “myImagesSEGMENTEDbreast” divided into normal and abnormal) to feed the convolutional neural network in Matlab2018a (“AlexCNNbreast.m” code to obtain the classification results and CNN models); the two CNN models from the 2-fold cross validation (“myNet_s1.mat” and “myNet_s2.mat”) that obtained 100% of TPR, SPC, and ACC; plotConfMat.m code to obtain the confusion matrices of CNN, TRF, MLP, and BN. Video results of Figures 3 and 4: VIDEO 1 of Figure 4 in mp4 format—this video describes the initial elliptical points for gradient vector flow using the curvature function k of right and left breasts; VIDEO 2 of Figure 4 in mp4 format—this video describes the gradient vector flow segmentation of the breast region of interest. WEKA breast data features: 155 × 63 classical features in Weka for TRF, MLP, and BN results. Run “BreastDatasetFeatures.arff” for obtaining the classification results. [file 9807619.f1.zip › 9807619_Addition_SupplementaryMaterials/MATLAB breast data/myImagesSEGMENTEDbreast/Abnormal/15.bmp]

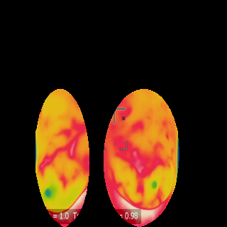

Supplement: Supplementary Materials — Figures 1–7: original images of Figures 1 to 7 in png format. MATLAB breast data: first run “AlexCNNbreast.m” code. Segmentation results of the breast images extracted from the gradient flow method (63 images in bmp format on folder “myImagesSEGMENTEDbreast” divided into normal and abnormal) to feed the convolutional neural network in Matlab2018a (“AlexCNNbreast.m” code to obtain the classification results and CNN models); the two CNN models from the 2-fold cross validation (“myNet_s1.mat” and “myNet_s2.mat”) that obtained 100% of TPR, SPC, and ACC; plotConfMat.m code to obtain the confusion matrices of CNN, TRF, MLP, and BN. Video results of Figures 3 and 4: VIDEO 1 of Figure 4 in mp4 format—this video describes the initial elliptical points for gradient vector flow using the curvature function k of right and left breasts; VIDEO 2 of Figure 4 in mp4 format—this video describes the gradient vector flow segmentation of the breast region of interest. WEKA breast data features: 155 × 63 classical features in Weka for TRF, MLP, and BN results. Run “BreastDatasetFeatures.arff” for obtaining the classification results. [file 9807619.f1.zip › 9807619_Addition_SupplementaryMaterials/MATLAB breast data/myImagesSEGMENTEDbreast/Abnormal/28.bmp]

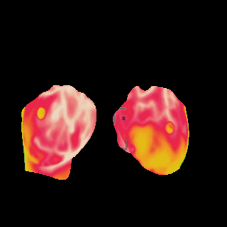

Supplement: Supplementary Materials — Figures 1–7: original images of Figures 1 to 7 in png format. MATLAB breast data: first run “AlexCNNbreast.m” code. Segmentation results of the breast images extracted from the gradient flow method (63 images in bmp format on folder “myImagesSEGMENTEDbreast” divided into normal and abnormal) to feed the convolutional neural network in Matlab2018a (“AlexCNNbreast.m” code to obtain the classification results and CNN models); the two CNN models from the 2-fold cross validation (“myNet_s1.mat” and “myNet_s2.mat”) that obtained 100% of TPR, SPC, and ACC; plotConfMat.m code to obtain the confusion matrices of CNN, TRF, MLP, and BN. Video results of Figures 3 and 4: VIDEO 1 of Figure 4 in mp4 format—this video describes the initial elliptical points for gradient vector flow using the curvature function k of right and left breasts; VIDEO 2 of Figure 4 in mp4 format—this video describes the gradient vector flow segmentation of the breast region of interest. WEKA breast data features: 155 × 63 classical features in Weka for TRF, MLP, and BN results. Run “BreastDatasetFeatures.arff” for obtaining the classification results. [file 9807619.f1.zip › 9807619_Addition_SupplementaryMaterials/MATLAB breast data/myImagesSEGMENTEDbreast/Abnormal/14.bmp]

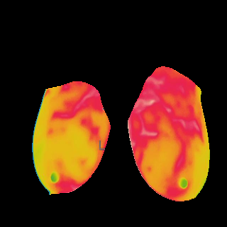

Supplement: Supplementary Materials — Figures 1–7: original images of Figures 1 to 7 in png format. MATLAB breast data: first run “AlexCNNbreast.m” code. Segmentation results of the breast images extracted from the gradient flow method (63 images in bmp format on folder “myImagesSEGMENTEDbreast” divided into normal and abnormal) to feed the convolutional neural network in Matlab2018a (“AlexCNNbreast.m” code to obtain the classification results and CNN models); the two CNN models from the 2-fold cross validation (“myNet_s1.mat” and “myNet_s2.mat”) that obtained 100% of TPR, SPC, and ACC; plotConfMat.m code to obtain the confusion matrices of CNN, TRF, MLP, and BN. Video results of Figures 3 and 4: VIDEO 1 of Figure 4 in mp4 format—this video describes the initial elliptical points for gradient vector flow using the curvature function k of right and left breasts; VIDEO 2 of Figure 4 in mp4 format—this video describes the gradient vector flow segmentation of the breast region of interest. WEKA breast data features: 155 × 63 classical features in Weka for TRF, MLP, and BN results. Run “BreastDatasetFeatures.arff” for obtaining the classification results. [file 9807619.f1.zip › 9807619_Addition_SupplementaryMaterials/MATLAB breast data/myImagesSEGMENTEDbreast/Abnormal/16.bmp]

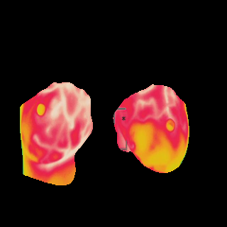

Supplement: Supplementary Materials — Figures 1–7: original images of Figures 1 to 7 in png format. MATLAB breast data: first run “AlexCNNbreast.m” code. Segmentation results of the breast images extracted from the gradient flow method (63 images in bmp format on folder “myImagesSEGMENTEDbreast” divided into normal and abnormal) to feed the convolutional neural network in Matlab2018a (“AlexCNNbreast.m” code to obtain the classification results and CNN models); the two CNN models from the 2-fold cross validation (“myNet_s1.mat” and “myNet_s2.mat”) that obtained 100% of TPR, SPC, and ACC; plotConfMat.m code to obtain the confusion matrices of CNN, TRF, MLP, and BN. Video results of Figures 3 and 4: VIDEO 1 of Figure 4 in mp4 format—this video describes the initial elliptical points for gradient vector flow using the curvature function k of right and left breasts; VIDEO 2 of Figure 4 in mp4 format—this video describes the gradient vector flow segmentation of the breast region of interest. WEKA breast data features: 155 × 63 classical features in Weka for TRF, MLP, and BN results. Run “BreastDatasetFeatures.arff” for obtaining the classification results. [file 9807619.f1.zip › 9807619_Addition_SupplementaryMaterials/MATLAB breast data/myImagesSEGMENTEDbreast/Abnormal/17.bmp]

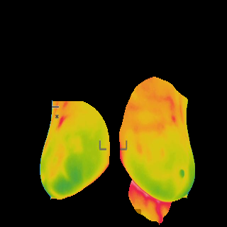

Supplement: Supplementary Materials — Figures 1–7: original images of Figures 1 to 7 in png format. MATLAB breast data: first run “AlexCNNbreast.m” code. Segmentation results of the breast images extracted from the gradient flow method (63 images in bmp format on folder “myImagesSEGMENTEDbreast” divided into normal and abnormal) to feed the convolutional neural network in Matlab2018a (“AlexCNNbreast.m” code to obtain the classification results and CNN models); the two CNN models from the 2-fold cross validation (“myNet_s1.mat” and “myNet_s2.mat”) that obtained 100% of TPR, SPC, and ACC; plotConfMat.m code to obtain the confusion matrices of CNN, TRF, MLP, and BN. Video results of Figures 3 and 4: VIDEO 1 of Figure 4 in mp4 format—this video describes the initial elliptical points for gradient vector flow using the curvature function k of right and left breasts; VIDEO 2 of Figure 4 in mp4 format—this video describes the gradient vector flow segmentation of the breast region of interest. WEKA breast data features: 155 × 63 classical features in Weka for TRF, MLP, and BN results. Run “BreastDatasetFeatures.arff” for obtaining the classification results. [file 9807619.f1.zip › 9807619_Addition_SupplementaryMaterials/MATLAB breast data/myImagesSEGMENTEDbreast/2NORMAL/26.bmp]

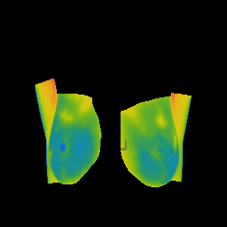

Supplement: Supplementary Materials — Figures 1–7: original images of Figures 1 to 7 in png format. MATLAB breast data: first run “AlexCNNbreast.m” code. Segmentation results of the breast images extracted from the gradient flow method (63 images in bmp format on folder “myImagesSEGMENTEDbreast” divided into normal and abnormal) to feed the convolutional neural network in Matlab2018a (“AlexCNNbreast.m” code to obtain the classification results and CNN models); the two CNN models from the 2-fold cross validation (“myNet_s1.mat” and “myNet_s2.mat”) that obtained 100% of TPR, SPC, and ACC; plotConfMat.m code to obtain the confusion matrices of CNN, TRF, MLP, and BN. Video results of Figures 3 and 4: VIDEO 1 of Figure 4 in mp4 format—this video describes the initial elliptical points for gradient vector flow using the curvature function k of right and left breasts; VIDEO 2 of Figure 4 in mp4 format—this video describes the gradient vector flow segmentation of the breast region of interest. WEKA breast data features: 155 × 63 classical features in Weka for TRF, MLP, and BN results. Run “BreastDatasetFeatures.arff” for obtaining the classification results. [file 9807619.f1.zip › 9807619_Addition_SupplementaryMaterials/MATLAB breast data/myImagesSEGMENTEDbreast/2NORMAL/32.bmp]

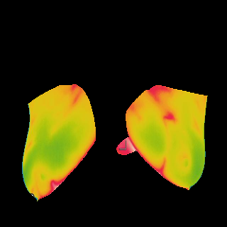

Supplement: Supplementary Materials — Figures 1–7: original images of Figures 1 to 7 in png format. MATLAB breast data: first run “AlexCNNbreast.m” code. Segmentation results of the breast images extracted from the gradient flow method (63 images in bmp format on folder “myImagesSEGMENTEDbreast” divided into normal and abnormal) to feed the convolutional neural network in Matlab2018a (“AlexCNNbreast.m” code to obtain the classification results and CNN models); the two CNN models from the 2-fold cross validation (“myNet_s1.mat” and “myNet_s2.mat”) that obtained 100% of TPR, SPC, and ACC; plotConfMat.m code to obtain the confusion matrices of CNN, TRF, MLP, and BN. Video results of Figures 3 and 4: VIDEO 1 of Figure 4 in mp4 format—this video describes the initial elliptical points for gradient vector flow using the curvature function k of right and left breasts; VIDEO 2 of Figure 4 in mp4 format—this video describes the gradient vector flow segmentation of the breast region of interest. WEKA breast data features: 155 × 63 classical features in Weka for TRF, MLP, and BN results. Run “BreastDatasetFeatures.arff” for obtaining the classification results. [file 9807619.f1.zip › 9807619_Addition_SupplementaryMaterials/MATLAB breast data/myImagesSEGMENTEDbreast/2NORMAL/33.bmp]

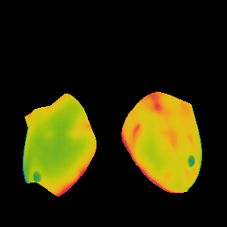

Supplement: Supplementary Materials — Figures 1–7: original images of Figures 1 to 7 in png format. MATLAB breast data: first run “AlexCNNbreast.m” code. Segmentation results of the breast images extracted from the gradient flow method (63 images in bmp format on folder “myImagesSEGMENTEDbreast” divided into normal and abnormal) to feed the convolutional neural network in Matlab2018a (“AlexCNNbreast.m” code to obtain the classification results and CNN models); the two CNN models from the 2-fold cross validation (“myNet_s1.mat” and “myNet_s2.mat”) that obtained 100% of TPR, SPC, and ACC; plotConfMat.m code to obtain the confusion matrices of CNN, TRF, MLP, and BN. Video results of Figures 3 and 4: VIDEO 1 of Figure 4 in mp4 format—this video describes the initial elliptical points for gradient vector flow using the curvature function k of right and left breasts; VIDEO 2 of Figure 4 in mp4 format—this video describes the gradient vector flow segmentation of the breast region of interest. WEKA breast data features: 155 × 63 classical features in Weka for TRF, MLP, and BN results. Run “BreastDatasetFeatures.arff” for obtaining the classification results. [file 9807619.f1.zip › 9807619_Addition_SupplementaryMaterials/MATLAB breast data/myImagesSEGMENTEDbreast/2NORMAL/27.bmp]

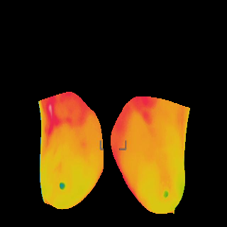

Supplement: Supplementary Materials — Figures 1–7: original images of Figures 1 to 7 in png format. MATLAB breast data: first run “AlexCNNbreast.m” code. Segmentation results of the breast images extracted from the gradient flow method (63 images in bmp format on folder “myImagesSEGMENTEDbreast” divided into normal and abnormal) to feed the convolutional neural network in Matlab2018a (“AlexCNNbreast.m” code to obtain the classification results and CNN models); the two CNN models from the 2-fold cross validation (“myNet_s1.mat” and “myNet_s2.mat”) that obtained 100% of TPR, SPC, and ACC; plotConfMat.m code to obtain the confusion matrices of CNN, TRF, MLP, and BN. Video results of Figures 3 and 4: VIDEO 1 of Figure 4 in mp4 format—this video describes the initial elliptical points for gradient vector flow using the curvature function k of right and left breasts; VIDEO 2 of Figure 4 in mp4 format—this video describes the gradient vector flow segmentation of the breast region of interest. WEKA breast data features: 155 × 63 classical features in Weka for TRF, MLP, and BN results. Run “BreastDatasetFeatures.arff” for obtaining the classification results. [file 9807619.f1.zip › 9807619_Addition_SupplementaryMaterials/MATLAB breast data/myImagesSEGMENTEDbreast/2NORMAL/19.bmp]

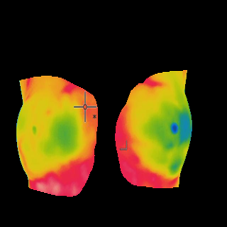

Supplement: Supplementary Materials — Figures 1–7: original images of Figures 1 to 7 in png format. MATLAB breast data: first run “AlexCNNbreast.m” code. Segmentation results of the breast images extracted from the gradient flow method (63 images in bmp format on folder “myImagesSEGMENTEDbreast” divided into normal and abnormal) to feed the convolutional neural network in Matlab2018a (“AlexCNNbreast.m” code to obtain the classification results and CNN models); the two CNN models from the 2-fold cross validation (“myNet_s1.mat” and “myNet_s2.mat”) that obtained 100% of TPR, SPC, and ACC; plotConfMat.m code to obtain the confusion matrices of CNN, TRF, MLP, and BN. Video results of Figures 3 and 4: VIDEO 1 of Figure 4 in mp4 format—this video describes the initial elliptical points for gradient vector flow using the curvature function k of right and left breasts; VIDEO 2 of Figure 4 in mp4 format—this video describes the gradient vector flow segmentation of the breast region of interest. WEKA breast data features: 155 × 63 classical features in Weka for TRF, MLP, and BN results. Run “BreastDatasetFeatures.arff” for obtaining the classification results. [file 9807619.f1.zip › 9807619_Addition_SupplementaryMaterials/MATLAB breast data/myImagesSEGMENTEDbreast/2NORMAL/31.bmp]

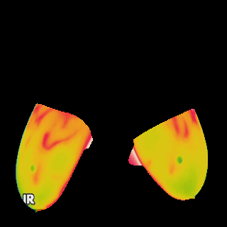

Supplement: Supplementary Materials — Figures 1–7: original images of Figures 1 to 7 in png format. MATLAB breast data: first run “AlexCNNbreast.m” code. Segmentation results of the breast images extracted from the gradient flow method (63 images in bmp format on folder “myImagesSEGMENTEDbreast” divided into normal and abnormal) to feed the convolutional neural network in Matlab2018a (“AlexCNNbreast.m” code to obtain the classification results and CNN models); the two CNN models from the 2-fold cross validation (“myNet_s1.mat” and “myNet_s2.mat”) that obtained 100% of TPR, SPC, and ACC; plotConfMat.m code to obtain the confusion matrices of CNN, TRF, MLP, and BN. Video results of Figures 3 and 4: VIDEO 1 of Figure 4 in mp4 format—this video describes the initial elliptical points for gradient vector flow using the curvature function k of right and left breasts; VIDEO 2 of Figure 4 in mp4 format—this video describes the gradient vector flow segmentation of the breast region of interest. WEKA breast data features: 155 × 63 classical features in Weka for TRF, MLP, and BN results. Run “BreastDatasetFeatures.arff” for obtaining the classification results. [file 9807619.f1.zip › 9807619_Addition_SupplementaryMaterials/MATLAB breast data/myImagesSEGMENTEDbreast/2NORMAL/25.bmp]

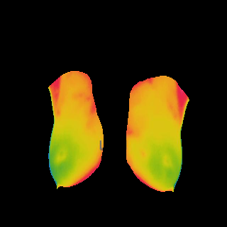

Supplement: Supplementary Materials — Figures 1–7: original images of Figures 1 to 7 in png format. MATLAB breast data: first run “AlexCNNbreast.m” code. Segmentation results of the breast images extracted from the gradient flow method (63 images in bmp format on folder “myImagesSEGMENTEDbreast” divided into normal and abnormal) to feed the convolutional neural network in Matlab2018a (“AlexCNNbreast.m” code to obtain the classification results and CNN models); the two CNN models from the 2-fold cross validation (“myNet_s1.mat” and “myNet_s2.mat”) that obtained 100% of TPR, SPC, and ACC; plotConfMat.m code to obtain the confusion matrices of CNN, TRF, MLP, and BN. Video results of Figures 3 and 4: VIDEO 1 of Figure 4 in mp4 format—this video describes the initial elliptical points for gradient vector flow using the curvature function k of right and left breasts; VIDEO 2 of Figure 4 in mp4 format—this video describes the gradient vector flow segmentation of the breast region of interest. WEKA breast data features: 155 × 63 classical features in Weka for TRF, MLP, and BN results. Run “BreastDatasetFeatures.arff” for obtaining the classification results. [file 9807619.f1.zip › 9807619_Addition_SupplementaryMaterials/MATLAB breast data/myImagesSEGMENTEDbreast/2NORMAL/24.bmp]

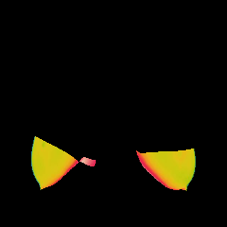

Supplement: Supplementary Materials — Figures 1–7: original images of Figures 1 to 7 in png format. MATLAB breast data: first run “AlexCNNbreast.m” code. Segmentation results of the breast images extracted from the gradient flow method (63 images in bmp format on folder “myImagesSEGMENTEDbreast” divided into normal and abnormal) to feed the convolutional neural network in Matlab2018a (“AlexCNNbreast.m” code to obtain the classification results and CNN models); the two CNN models from the 2-fold cross validation (“myNet_s1.mat” and “myNet_s2.mat”) that obtained 100% of TPR, SPC, and ACC; plotConfMat.m code to obtain the confusion matrices of CNN, TRF, MLP, and BN. Video results of Figures 3 and 4: VIDEO 1 of Figure 4 in mp4 format—this video describes the initial elliptical points for gradient vector flow using the curvature function k of right and left breasts; VIDEO 2 of Figure 4 in mp4 format—this video describes the gradient vector flow segmentation of the breast region of interest. WEKA breast data features: 155 × 63 classical features in Weka for TRF, MLP, and BN results. Run “BreastDatasetFeatures.arff” for obtaining the classification results. [file 9807619.f1.zip › 9807619_Addition_SupplementaryMaterials/MATLAB breast data/myImagesSEGMENTEDbreast/2NORMAL/30.bmp]

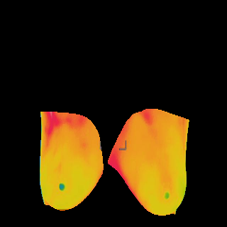

Supplement: Supplementary Materials — Figures 1–7: original images of Figures 1 to 7 in png format. MATLAB breast data: first run “AlexCNNbreast.m” code. Segmentation results of the breast images extracted from the gradient flow method (63 images in bmp format on folder “myImagesSEGMENTEDbreast” divided into normal and abnormal) to feed the convolutional neural network in Matlab2018a (“AlexCNNbreast.m” code to obtain the classification results and CNN models); the two CNN models from the 2-fold cross validation (“myNet_s1.mat” and “myNet_s2.mat”) that obtained 100% of TPR, SPC, and ACC; plotConfMat.m code to obtain the confusion matrices of CNN, TRF, MLP, and BN. Video results of Figures 3 and 4: VIDEO 1 of Figure 4 in mp4 format—this video describes the initial elliptical points for gradient vector flow using the curvature function k of right and left breasts; VIDEO 2 of Figure 4 in mp4 format—this video describes the gradient vector flow segmentation of the breast region of interest. WEKA breast data features: 155 × 63 classical features in Weka for TRF, MLP, and BN results. Run “BreastDatasetFeatures.arff” for obtaining the classification results. [file 9807619.f1.zip › 9807619_Addition_SupplementaryMaterials/MATLAB breast data/myImagesSEGMENTEDbreast/2NORMAL/18.bmp]

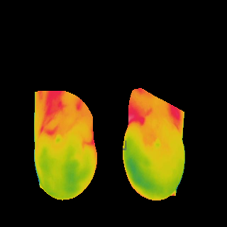

Supplement: Supplementary Materials — Figures 1–7: original images of Figures 1 to 7 in png format. MATLAB breast data: first run “AlexCNNbreast.m” code. Segmentation results of the breast images extracted from the gradient flow method (63 images in bmp format on folder “myImagesSEGMENTEDbreast” divided into normal and abnormal) to feed the convolutional neural network in Matlab2018a (“AlexCNNbreast.m” code to obtain the classification results and CNN models); the two CNN models from the 2-fold cross validation (“myNet_s1.mat” and “myNet_s2.mat”) that obtained 100% of TPR, SPC, and ACC; plotConfMat.m code to obtain the confusion matrices of CNN, TRF, MLP, and BN. Video results of Figures 3 and 4: VIDEO 1 of Figure 4 in mp4 format—this video describes the initial elliptical points for gradient vector flow using the curvature function k of right and left breasts; VIDEO 2 of Figure 4 in mp4 format—this video describes the gradient vector flow segmentation of the breast region of interest. WEKA breast data features: 155 × 63 classical features in Weka for TRF, MLP, and BN results. Run “BreastDatasetFeatures.arff” for obtaining the classification results. [file 9807619.f1.zip › 9807619_Addition_SupplementaryMaterials/MATLAB breast data/myImagesSEGMENTEDbreast/2NORMAL/34.bmp]

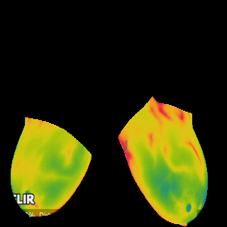

Supplement: Supplementary Materials — Figures 1–7: original images of Figures 1 to 7 in png format. MATLAB breast data: first run “AlexCNNbreast.m” code. Segmentation results of the breast images extracted from the gradient flow method (63 images in bmp format on folder “myImagesSEGMENTEDbreast” divided into normal and abnormal) to feed the convolutional neural network in Matlab2018a (“AlexCNNbreast.m” code to obtain the classification results and CNN models); the two CNN models from the 2-fold cross validation (“myNet_s1.mat” and “myNet_s2.mat”) that obtained 100% of TPR, SPC, and ACC; plotConfMat.m code to obtain the confusion matrices of CNN, TRF, MLP, and BN. Video results of Figures 3 and 4: VIDEO 1 of Figure 4 in mp4 format—this video describes the initial elliptical points for gradient vector flow using the curvature function k of right and left breasts; VIDEO 2 of Figure 4 in mp4 format—this video describes the gradient vector flow segmentation of the breast region of interest. WEKA breast data features: 155 × 63 classical features in Weka for TRF, MLP, and BN results. Run “BreastDatasetFeatures.arff” for obtaining the classification results. [file 9807619.f1.zip › 9807619_Addition_SupplementaryMaterials/MATLAB breast data/myImagesSEGMENTEDbreast/2NORMAL/20.bmp]

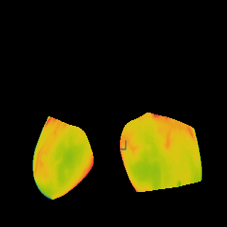

Supplement: Supplementary Materials — Figures 1–7: original images of Figures 1 to 7 in png format. MATLAB breast data: first run “AlexCNNbreast.m” code. Segmentation results of the breast images extracted from the gradient flow method (63 images in bmp format on folder “myImagesSEGMENTEDbreast” divided into normal and abnormal) to feed the convolutional neural network in Matlab2018a (“AlexCNNbreast.m” code to obtain the classification results and CNN models); the two CNN models from the 2-fold cross validation (“myNet_s1.mat” and “myNet_s2.mat”) that obtained 100% of TPR, SPC, and ACC; plotConfMat.m code to obtain the confusion matrices of CNN, TRF, MLP, and BN. Video results of Figures 3 and 4: VIDEO 1 of Figure 4 in mp4 format—this video describes the initial elliptical points for gradient vector flow using the curvature function k of right and left breasts; VIDEO 2 of Figure 4 in mp4 format—this video describes the gradient vector flow segmentation of the breast region of interest. WEKA breast data features: 155 × 63 classical features in Weka for TRF, MLP, and BN results. Run “BreastDatasetFeatures.arff” for obtaining the classification results. [file 9807619.f1.zip › 9807619_Addition_SupplementaryMaterials/MATLAB breast data/myImagesSEGMENTEDbreast/2NORMAL/21.bmp]

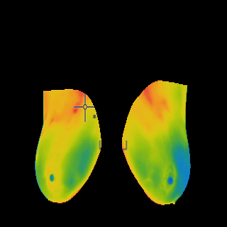

Supplement: Supplementary Materials — Figures 1–7: original images of Figures 1 to 7 in png format. MATLAB breast data: first run “AlexCNNbreast.m” code. Segmentation results of the breast images extracted from the gradient flow method (63 images in bmp format on folder “myImagesSEGMENTEDbreast” divided into normal and abnormal) to feed the convolutional neural network in Matlab2018a (“AlexCNNbreast.m” code to obtain the classification results and CNN models); the two CNN models from the 2-fold cross validation (“myNet_s1.mat” and “myNet_s2.mat”) that obtained 100% of TPR, SPC, and ACC; plotConfMat.m code to obtain the confusion matrices of CNN, TRF, MLP, and BN. Video results of Figures 3 and 4: VIDEO 1 of Figure 4 in mp4 format—this video describes the initial elliptical points for gradient vector flow using the curvature function k of right and left breasts; VIDEO 2 of Figure 4 in mp4 format—this video describes the gradient vector flow segmentation of the breast region of interest. WEKA breast data features: 155 × 63 classical features in Weka for TRF, MLP, and BN results. Run “BreastDatasetFeatures.arff” for obtaining the classification results. [file 9807619.f1.zip › 9807619_Addition_SupplementaryMaterials/MATLAB breast data/myImagesSEGMENTEDbreast/2NORMAL/35.bmp]

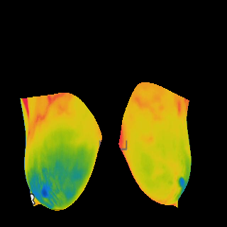

Supplement: Supplementary Materials — Figures 1–7: original images of Figures 1 to 7 in png format. MATLAB breast data: first run “AlexCNNbreast.m” code. Segmentation results of the breast images extracted from the gradient flow method (63 images in bmp format on folder “myImagesSEGMENTEDbreast” divided into normal and abnormal) to feed the convolutional neural network in Matlab2018a (“AlexCNNbreast.m” code to obtain the classification results and CNN models); the two CNN models from the 2-fold cross validation (“myNet_s1.mat” and “myNet_s2.mat”) that obtained 100% of TPR, SPC, and ACC; plotConfMat.m code to obtain the confusion matrices of CNN, TRF, MLP, and BN. Video results of Figures 3 and 4: VIDEO 1 of Figure 4 in mp4 format—this video describes the initial elliptical points for gradient vector flow using the curvature function k of right and left breasts; VIDEO 2 of Figure 4 in mp4 format—this video describes the gradient vector flow segmentation of the breast region of interest. WEKA breast data features: 155 × 63 classical features in Weka for TRF, MLP, and BN results. Run “BreastDatasetFeatures.arff” for obtaining the classification results. [file 9807619.f1.zip › 9807619_Addition_SupplementaryMaterials/MATLAB breast data/myImagesSEGMENTEDbreast/2NORMAL/23.bmp]

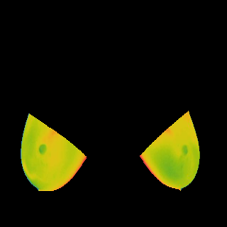

Supplement: Supplementary Materials — Figures 1–7: original images of Figures 1 to 7 in png format. MATLAB breast data: first run “AlexCNNbreast.m” code. Segmentation results of the breast images extracted from the gradient flow method (63 images in bmp format on folder “myImagesSEGMENTEDbreast” divided into normal and abnormal) to feed the convolutional neural network in Matlab2018a (“AlexCNNbreast.m” code to obtain the classification results and CNN models); the two CNN models from the 2-fold cross validation (“myNet_s1.mat” and “myNet_s2.mat”) that obtained 100% of TPR, SPC, and ACC; plotConfMat.m code to obtain the confusion matrices of CNN, TRF, MLP, and BN. Video results of Figures 3 and 4: VIDEO 1 of Figure 4 in mp4 format—this video describes the initial elliptical points for gradient vector flow using the curvature function k of right and left breasts; VIDEO 2 of Figure 4 in mp4 format—this video describes the gradient vector flow segmentation of the breast region of interest. WEKA breast data features: 155 × 63 classical features in Weka for TRF, MLP, and BN results. Run “BreastDatasetFeatures.arff” for obtaining the classification results. [file 9807619.f1.zip › 9807619_Addition_SupplementaryMaterials/MATLAB breast data/myImagesSEGMENTEDbreast/2NORMAL/22.bmp]

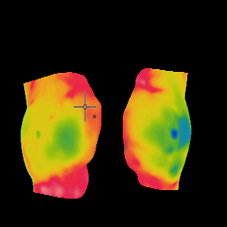

Supplement: Supplementary Materials — Figures 1–7: original images of Figures 1 to 7 in png format. MATLAB breast data: first run “AlexCNNbreast.m” code. Segmentation results of the breast images extracted from the gradient flow method (63 images in bmp format on folder “myImagesSEGMENTEDbreast” divided into normal and abnormal) to feed the convolutional neural network in Matlab2018a (“AlexCNNbreast.m” code to obtain the classification results and CNN models); the two CNN models from the 2-fold cross validation (“myNet_s1.mat” and “myNet_s2.mat”) that obtained 100% of TPR, SPC, and ACC; plotConfMat.m code to obtain the confusion matrices of CNN, TRF, MLP, and BN. Video results of Figures 3 and 4: VIDEO 1 of Figure 4 in mp4 format—this video describes the initial elliptical points for gradient vector flow using the curvature function k of right and left breasts; VIDEO 2 of Figure 4 in mp4 format—this video describes the gradient vector flow segmentation of the breast region of interest. WEKA breast data features: 155 × 63 classical features in Weka for TRF, MLP, and BN results. Run “BreastDatasetFeatures.arff” for obtaining the classification results. [file 9807619.f1.zip › 9807619_Addition_SupplementaryMaterials/MATLAB breast data/myImagesSEGMENTEDbreast/2NORMAL/3.bmp]

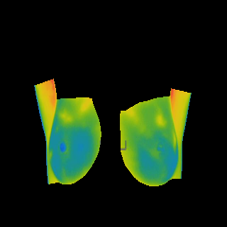

Supplement: Supplementary Materials — Figures 1–7: original images of Figures 1 to 7 in png format. MATLAB breast data: first run “AlexCNNbreast.m” code. Segmentation results of the breast images extracted from the gradient flow method (63 images in bmp format on folder “myImagesSEGMENTEDbreast” divided into normal and abnormal) to feed the convolutional neural network in Matlab2018a (“AlexCNNbreast.m” code to obtain the classification results and CNN models); the two CNN models from the 2-fold cross validation (“myNet_s1.mat” and “myNet_s2.mat”) that obtained 100% of TPR, SPC, and ACC; plotConfMat.m code to obtain the confusion matrices of CNN, TRF, MLP, and BN. Video results of Figures 3 and 4: VIDEO 1 of Figure 4 in mp4 format—this video describes the initial elliptical points for gradient vector flow using the curvature function k of right and left breasts; VIDEO 2 of Figure 4 in mp4 format—this video describes the gradient vector flow segmentation of the breast region of interest. WEKA breast data features: 155 × 63 classical features in Weka for TRF, MLP, and BN results. Run “BreastDatasetFeatures.arff” for obtaining the classification results. [file 9807619.f1.zip › 9807619_Addition_SupplementaryMaterials/MATLAB breast data/myImagesSEGMENTEDbreast/2NORMAL/2.bmp]

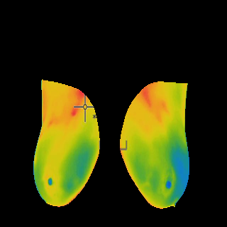

Supplement: Supplementary Materials — Figures 1–7: original images of Figures 1 to 7 in png format. MATLAB breast data: first run “AlexCNNbreast.m” code. Segmentation results of the breast images extracted from the gradient flow method (63 images in bmp format on folder “myImagesSEGMENTEDbreast” divided into normal and abnormal) to feed the convolutional neural network in Matlab2018a (“AlexCNNbreast.m” code to obtain the classification results and CNN models); the two CNN models from the 2-fold cross validation (“myNet_s1.mat” and “myNet_s2.mat”) that obtained 100% of TPR, SPC, and ACC; plotConfMat.m code to obtain the confusion matrices of CNN, TRF, MLP, and BN. Video results of Figures 3 and 4: VIDEO 1 of Figure 4 in mp4 format—this video describes the initial elliptical points for gradient vector flow using the curvature function k of right and left breasts; VIDEO 2 of Figure 4 in mp4 format—this video describes the gradient vector flow segmentation of the breast region of interest. WEKA breast data features: 155 × 63 classical features in Weka for TRF, MLP, and BN results. Run “BreastDatasetFeatures.arff” for obtaining the classification results. [file 9807619.f1.zip › 9807619_Addition_SupplementaryMaterials/MATLAB breast data/myImagesSEGMENTEDbreast/2NORMAL/1.bmp]

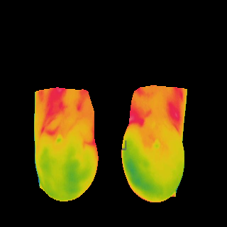

Supplement: Supplementary Materials — Figures 1–7: original images of Figures 1 to 7 in png format. MATLAB breast data: first run “AlexCNNbreast.m” code. Segmentation results of the breast images extracted from the gradient flow method (63 images in bmp format on folder “myImagesSEGMENTEDbreast” divided into normal and abnormal) to feed the convolutional neural network in Matlab2018a (“AlexCNNbreast.m” code to obtain the classification results and CNN models); the two CNN models from the 2-fold cross validation (“myNet_s1.mat” and “myNet_s2.mat”) that obtained 100% of TPR, SPC, and ACC; plotConfMat.m code to obtain the confusion matrices of CNN, TRF, MLP, and BN. Video results of Figures 3 and 4: VIDEO 1 of Figure 4 in mp4 format—this video describes the initial elliptical points for gradient vector flow using the curvature function k of right and left breasts; VIDEO 2 of Figure 4 in mp4 format—this video describes the gradient vector flow segmentation of the breast region of interest. WEKA breast data features: 155 × 63 classical features in Weka for TRF, MLP, and BN results. Run “BreastDatasetFeatures.arff” for obtaining the classification results. [file 9807619.f1.zip › 9807619_Addition_SupplementaryMaterials/MATLAB breast data/myImagesSEGMENTEDbreast/2NORMAL/5.bmp]

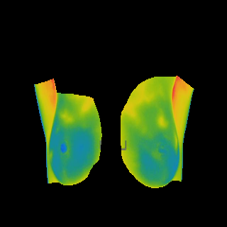

Supplement: Supplementary Materials — Figures 1–7: original images of Figures 1 to 7 in png format. MATLAB breast data: first run “AlexCNNbreast.m” code. Segmentation results of the breast images extracted from the gradient flow method (63 images in bmp format on folder “myImagesSEGMENTEDbreast” divided into normal and abnormal) to feed the convolutional neural network in Matlab2018a (“AlexCNNbreast.m” code to obtain the classification results and CNN models); the two CNN models from the 2-fold cross validation (“myNet_s1.mat” and “myNet_s2.mat”) that obtained 100% of TPR, SPC, and ACC; plotConfMat.m code to obtain the confusion matrices of CNN, TRF, MLP, and BN. Video results of Figures 3 and 4: VIDEO 1 of Figure 4 in mp4 format—this video describes the initial elliptical points for gradient vector flow using the curvature function k of right and left breasts; VIDEO 2 of Figure 4 in mp4 format—this video describes the gradient vector flow segmentation of the breast region of interest. WEKA breast data features: 155 × 63 classical features in Weka for TRF, MLP, and BN results. Run “BreastDatasetFeatures.arff” for obtaining the classification results. [file 9807619.f1.zip › 9807619_Addition_SupplementaryMaterials/MATLAB breast data/myImagesSEGMENTEDbreast/2NORMAL/4.bmp]

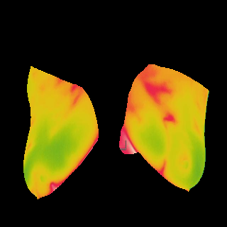

Supplement: Supplementary Materials — Figures 1–7: original images of Figures 1 to 7 in png format. MATLAB breast data: first run “AlexCNNbreast.m” code. Segmentation results of the breast images extracted from the gradient flow method (63 images in bmp format on folder “myImagesSEGMENTEDbreast” divided into normal and abnormal) to feed the convolutional neural network in Matlab2018a (“AlexCNNbreast.m” code to obtain the classification results and CNN models); the two CNN models from the 2-fold cross validation (“myNet_s1.mat” and “myNet_s2.mat”) that obtained 100% of TPR, SPC, and ACC; plotConfMat.m code to obtain the confusion matrices of CNN, TRF, MLP, and BN. Video results of Figures 3 and 4: VIDEO 1 of Figure 4 in mp4 format—this video describes the initial elliptical points for gradient vector flow using the curvature function k of right and left breasts; VIDEO 2 of Figure 4 in mp4 format—this video describes the gradient vector flow segmentation of the breast region of interest. WEKA breast data features: 155 × 63 classical features in Weka for TRF, MLP, and BN results. Run “BreastDatasetFeatures.arff” for obtaining the classification results. [file 9807619.f1.zip › 9807619_Addition_SupplementaryMaterials/MATLAB breast data/myImagesSEGMENTEDbreast/2NORMAL/6.bmp]

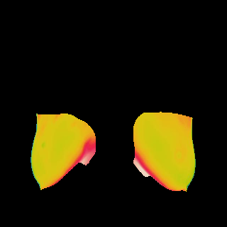

Supplement: Supplementary Materials — Figures 1–7: original images of Figures 1 to 7 in png format. MATLAB breast data: first run “AlexCNNbreast.m” code. Segmentation results of the breast images extracted from the gradient flow method (63 images in bmp format on folder “myImagesSEGMENTEDbreast” divided into normal and abnormal) to feed the convolutional neural network in Matlab2018a (“AlexCNNbreast.m” code to obtain the classification results and CNN models); the two CNN models from the 2-fold cross validation (“myNet_s1.mat” and “myNet_s2.mat”) that obtained 100% of TPR, SPC, and ACC; plotConfMat.m code to obtain the confusion matrices of CNN, TRF, MLP, and BN. Video results of Figures 3 and 4: VIDEO 1 of Figure 4 in mp4 format—this video describes the initial elliptical points for gradient vector flow using the curvature function k of right and left breasts; VIDEO 2 of Figure 4 in mp4 format—this video describes the gradient vector flow segmentation of the breast region of interest. WEKA breast data features: 155 × 63 classical features in Weka for TRF, MLP, and BN results. Run “BreastDatasetFeatures.arff” for obtaining the classification results. [file 9807619.f1.zip › 9807619_Addition_SupplementaryMaterials/MATLAB breast data/myImagesSEGMENTEDbreast/2NORMAL/7.bmp]

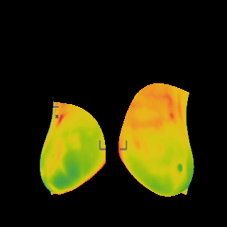

Supplement: Supplementary Materials — Figures 1–7: original images of Figures 1 to 7 in png format. MATLAB breast data: first run “AlexCNNbreast.m” code. Segmentation results of the breast images extracted from the gradient flow method (63 images in bmp format on folder “myImagesSEGMENTEDbreast” divided into normal and abnormal) to feed the convolutional neural network in Matlab2018a (“AlexCNNbreast.m” code to obtain the classification results and CNN models); the two CNN models from the 2-fold cross validation (“myNet_s1.mat” and “myNet_s2.mat”) that obtained 100% of TPR, SPC, and ACC; plotConfMat.m code to obtain the confusion matrices of CNN, TRF, MLP, and BN. Video results of Figures 3 and 4: VIDEO 1 of Figure 4 in mp4 format—this video describes the initial elliptical points for gradient vector flow using the curvature function k of right and left breasts; VIDEO 2 of Figure 4 in mp4 format—this video describes the gradient vector flow segmentation of the breast region of interest. WEKA breast data features: 155 × 63 classical features in Weka for TRF, MLP, and BN results. Run “BreastDatasetFeatures.arff” for obtaining the classification results. [file 9807619.f1.zip › 9807619_Addition_SupplementaryMaterials/MATLAB breast data/myImagesSEGMENTEDbreast/2NORMAL/9.bmp]

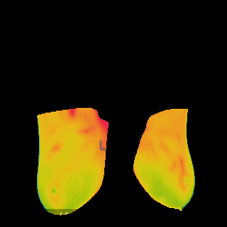

Supplement: Supplementary Materials — Figures 1–7: original images of Figures 1 to 7 in png format. MATLAB breast data: first run “AlexCNNbreast.m” code. Segmentation results of the breast images extracted from the gradient flow method (63 images in bmp format on folder “myImagesSEGMENTEDbreast” divided into normal and abnormal) to feed the convolutional neural network in Matlab2018a (“AlexCNNbreast.m” code to obtain the classification results and CNN models); the two CNN models from the 2-fold cross validation (“myNet_s1.mat” and “myNet_s2.mat”) that obtained 100% of TPR, SPC, and ACC; plotConfMat.m code to obtain the confusion matrices of CNN, TRF, MLP, and BN. Video results of Figures 3 and 4: VIDEO 1 of Figure 4 in mp4 format—this video describes the initial elliptical points for gradient vector flow using the curvature function k of right and left breasts; VIDEO 2 of Figure 4 in mp4 format—this video describes the gradient vector flow segmentation of the breast region of interest. WEKA breast data features: 155 × 63 classical features in Weka for TRF, MLP, and BN results. Run “BreastDatasetFeatures.arff” for obtaining the classification results. [file 9807619.f1.zip › 9807619_Addition_SupplementaryMaterials/MATLAB breast data/myImagesSEGMENTEDbreast/2NORMAL/8.bmp]

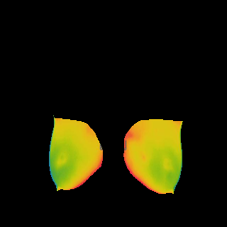

Supplement: Supplementary Materials — Figures 1–7: original images of Figures 1 to 7 in png format. MATLAB breast data: first run “AlexCNNbreast.m” code. Segmentation results of the breast images extracted from the gradient flow method (63 images in bmp format on folder “myImagesSEGMENTEDbreast” divided into normal and abnormal) to feed the convolutional neural network in Matlab2018a (“AlexCNNbreast.m” code to obtain the classification results and CNN models); the two CNN models from the 2-fold cross validation (“myNet_s1.mat” and “myNet_s2.mat”) that obtained 100% of TPR, SPC, and ACC; plotConfMat.m code to obtain the confusion matrices of CNN, TRF, MLP, and BN. Video results of Figures 3 and 4: VIDEO 1 of Figure 4 in mp4 format—this video describes the initial elliptical points for gradient vector flow using the curvature function k of right and left breasts; VIDEO 2 of Figure 4 in mp4 format—this video describes the gradient vector flow segmentation of the breast region of interest. WEKA breast data features: 155 × 63 classical features in Weka for TRF, MLP, and BN results. Run “BreastDatasetFeatures.arff” for obtaining the classification results. [file 9807619.f1.zip › 9807619_Addition_SupplementaryMaterials/MATLAB breast data/myImagesSEGMENTEDbreast/2NORMAL/13.bmp]

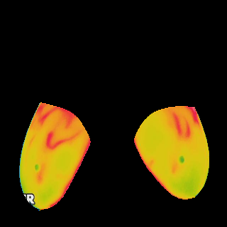

Supplement: Supplementary Materials — Figures 1–7: original images of Figures 1 to 7 in png format. MATLAB breast data: first run “AlexCNNbreast.m” code. Segmentation results of the breast images extracted from the gradient flow method (63 images in bmp format on folder “myImagesSEGMENTEDbreast” divided into normal and abnormal) to feed the convolutional neural network in Matlab2018a (“AlexCNNbreast.m” code to obtain the classification results and CNN models); the two CNN models from the 2-fold cross validation (“myNet_s1.mat” and “myNet_s2.mat”) that obtained 100% of TPR, SPC, and ACC; plotConfMat.m code to obtain the confusion matrices of CNN, TRF, MLP, and BN. Video results of Figures 3 and 4: VIDEO 1 of Figure 4 in mp4 format—this video describes the initial elliptical points for gradient vector flow using the curvature function k of right and left breasts; VIDEO 2 of Figure 4 in mp4 format—this video describes the gradient vector flow segmentation of the breast region of interest. WEKA breast data features: 155 × 63 classical features in Weka for TRF, MLP, and BN results. Run “BreastDatasetFeatures.arff” for obtaining the classification results. [file 9807619.f1.zip › 9807619_Addition_SupplementaryMaterials/MATLAB breast data/myImagesSEGMENTEDbreast/2NORMAL/12.bmp]

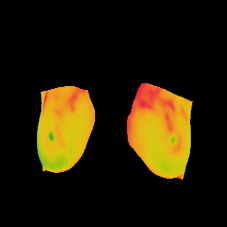

Supplement: Supplementary Materials — Figures 1–7: original images of Figures 1 to 7 in png format. MATLAB breast data: first run “AlexCNNbreast.m” code. Segmentation results of the breast images extracted from the gradient flow method (63 images in bmp format on folder “myImagesSEGMENTEDbreast” divided into normal and abnormal) to feed the convolutional neural network in Matlab2018a (“AlexCNNbreast.m” code to obtain the classification results and CNN models); the two CNN models from the 2-fold cross validation (“myNet_s1.mat” and “myNet_s2.mat”) that obtained 100% of TPR, SPC, and ACC; plotConfMat.m code to obtain the confusion matrices of CNN, TRF, MLP, and BN. Video results of Figures 3 and 4: VIDEO 1 of Figure 4 in mp4 format—this video describes the initial elliptical points for gradient vector flow using the curvature function k of right and left breasts; VIDEO 2 of Figure 4 in mp4 format—this video describes the gradient vector flow segmentation of the breast region of interest. WEKA breast data features: 155 × 63 classical features in Weka for TRF, MLP, and BN results. Run “BreastDatasetFeatures.arff” for obtaining the classification results. [file 9807619.f1.zip › 9807619_Addition_SupplementaryMaterials/MATLAB breast data/myImagesSEGMENTEDbreast/2NORMAL/10.bmp]

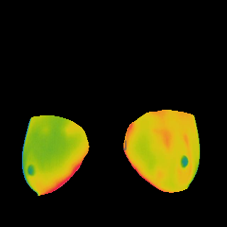

Supplement: Supplementary Materials — Figures 1–7: original images of Figures 1 to 7 in png format. MATLAB breast data: first run “AlexCNNbreast.m” code. Segmentation results of the breast images extracted from the gradient flow method (63 images in bmp format on folder “myImagesSEGMENTEDbreast” divided into normal and abnormal) to feed the convolutional neural network in Matlab2018a (“AlexCNNbreast.m” code to obtain the classification results and CNN models); the two CNN models from the 2-fold cross validation (“myNet_s1.mat” and “myNet_s2.mat”) that obtained 100% of TPR, SPC, and ACC; plotConfMat.m code to obtain the confusion matrices of CNN, TRF, MLP, and BN. Video results of Figures 3 and 4: VIDEO 1 of Figure 4 in mp4 format—this video describes the initial elliptical points for gradient vector flow using the curvature function k of right and left breasts; VIDEO 2 of Figure 4 in mp4 format—this video describes the gradient vector flow segmentation of the breast region of interest. WEKA breast data features: 155 × 63 classical features in Weka for TRF, MLP, and BN results. Run “BreastDatasetFeatures.arff” for obtaining the classification results. [file 9807619.f1.zip › 9807619_Addition_SupplementaryMaterials/MATLAB breast data/myImagesSEGMENTEDbreast/2NORMAL/11.bmp]

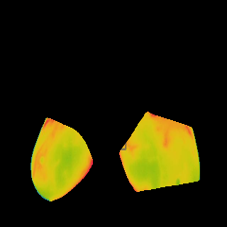

Supplement: Supplementary Materials — Figures 1–7: original images of Figures 1 to 7 in png format. MATLAB breast data: first run “AlexCNNbreast.m” code. Segmentation results of the breast images extracted from the gradient flow method (63 images in bmp format on folder “myImagesSEGMENTEDbreast” divided into normal and abnormal) to feed the convolutional neural network in Matlab2018a (“AlexCNNbreast.m” code to obtain the classification results and CNN models); the two CNN models from the 2-fold cross validation (“myNet_s1.mat” and “myNet_s2.mat”) that obtained 100% of TPR, SPC, and ACC; plotConfMat.m code to obtain the confusion matrices of CNN, TRF, MLP, and BN. Video results of Figures 3 and 4: VIDEO 1 of Figure 4 in mp4 format—this video describes the initial elliptical points for gradient vector flow using the curvature function k of right and left breasts; VIDEO 2 of Figure 4 in mp4 format—this video describes the gradient vector flow segmentation of the breast region of interest. WEKA breast data features: 155 × 63 classical features in Weka for TRF, MLP, and BN results. Run “BreastDatasetFeatures.arff” for obtaining the classification results. [file 9807619.f1.zip › 9807619_Addition_SupplementaryMaterials/MATLAB breast data/myImagesSEGMENTEDbreast/2NORMAL/15.bmp]

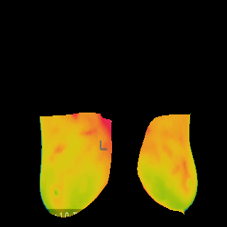

Supplement: Supplementary Materials — Figures 1–7: original images of Figures 1 to 7 in png format. MATLAB breast data: first run “AlexCNNbreast.m” code. Segmentation results of the breast images extracted from the gradient flow method (63 images in bmp format on folder “myImagesSEGMENTEDbreast” divided into normal and abnormal) to feed the convolutional neural network in Matlab2018a (“AlexCNNbreast.m” code to obtain the classification results and CNN models); the two CNN models from the 2-fold cross validation (“myNet_s1.mat” and “myNet_s2.mat”) that obtained 100% of TPR, SPC, and ACC; plotConfMat.m code to obtain the confusion matrices of CNN, TRF, MLP, and BN. Video results of Figures 3 and 4: VIDEO 1 of Figure 4 in mp4 format—this video describes the initial elliptical points for gradient vector flow using the curvature function k of right and left breasts; VIDEO 2 of Figure 4 in mp4 format—this video describes the gradient vector flow segmentation of the breast region of interest. WEKA breast data features: 155 × 63 classical features in Weka for TRF, MLP, and BN results. Run “BreastDatasetFeatures.arff” for obtaining the classification results. [file 9807619.f1.zip › 9807619_Addition_SupplementaryMaterials/MATLAB breast data/myImagesSEGMENTEDbreast/2NORMAL/29.bmp]

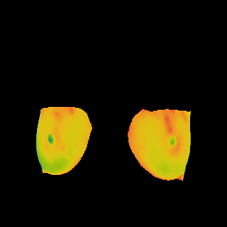

Supplement: Supplementary Materials — Figures 1–7: original images of Figures 1 to 7 in png format. MATLAB breast data: first run “AlexCNNbreast.m” code. Segmentation results of the breast images extracted from the gradient flow method (63 images in bmp format on folder “myImagesSEGMENTEDbreast” divided into normal and abnormal) to feed the convolutional neural network in Matlab2018a (“AlexCNNbreast.m” code to obtain the classification results and CNN models); the two CNN models from the 2-fold cross validation (“myNet_s1.mat” and “myNet_s2.mat”) that obtained 100% of TPR, SPC, and ACC; plotConfMat.m code to obtain the confusion matrices of CNN, TRF, MLP, and BN. Video results of Figures 3 and 4: VIDEO 1 of Figure 4 in mp4 format—this video describes the initial elliptical points for gradient vector flow using the curvature function k of right and left breasts; VIDEO 2 of Figure 4 in mp4 format—this video describes the gradient vector flow segmentation of the breast region of interest. WEKA breast data features: 155 × 63 classical features in Weka for TRF, MLP, and BN results. Run “BreastDatasetFeatures.arff” for obtaining the classification results. [file 9807619.f1.zip › 9807619_Addition_SupplementaryMaterials/MATLAB breast data/myImagesSEGMENTEDbreast/2NORMAL/28.bmp]

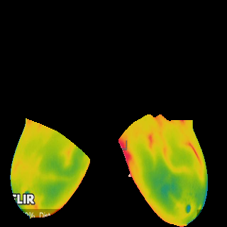

Supplement: Supplementary Materials — Figures 1–7: original images of Figures 1 to 7 in png format. MATLAB breast data: first run “AlexCNNbreast.m” code. Segmentation results of the breast images extracted from the gradient flow method (63 images in bmp format on folder “myImagesSEGMENTEDbreast” divided into normal and abnormal) to feed the convolutional neural network in Matlab2018a (“AlexCNNbreast.m” code to obtain the classification results and CNN models); the two CNN models from the 2-fold cross validation (“myNet_s1.mat” and “myNet_s2.mat”) that obtained 100% of TPR, SPC, and ACC; plotConfMat.m code to obtain the confusion matrices of CNN, TRF, MLP, and BN. Video results of Figures 3 and 4: VIDEO 1 of Figure 4 in mp4 format—this video describes the initial elliptical points for gradient vector flow using the curvature function k of right and left breasts; VIDEO 2 of Figure 4 in mp4 format—this video describes the gradient vector flow segmentation of the breast region of interest. WEKA breast data features: 155 × 63 classical features in Weka for TRF, MLP, and BN results. Run “BreastDatasetFeatures.arff” for obtaining the classification results. [file 9807619.f1.zip › 9807619_Addition_SupplementaryMaterials/MATLAB breast data/myImagesSEGMENTEDbreast/2NORMAL/14.bmp]

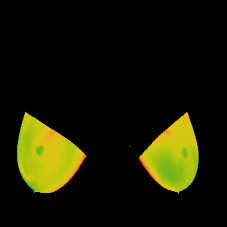

Supplement: Supplementary Materials — Figures 1–7: original images of Figures 1 to 7 in png format. MATLAB breast data: first run “AlexCNNbreast.m” code. Segmentation results of the breast images extracted from the gradient flow method (63 images in bmp format on folder “myImagesSEGMENTEDbreast” divided into normal and abnormal) to feed the convolutional neural network in Matlab2018a (“AlexCNNbreast.m” code to obtain the classification results and CNN models); the two CNN models from the 2-fold cross validation (“myNet_s1.mat” and “myNet_s2.mat”) that obtained 100% of TPR, SPC, and ACC; plotConfMat.m code to obtain the confusion matrices of CNN, TRF, MLP, and BN. Video results of Figures 3 and 4: VIDEO 1 of Figure 4 in mp4 format—this video describes the initial elliptical points for gradient vector flow using the curvature function k of right and left breasts; VIDEO 2 of Figure 4 in mp4 format—this video describes the gradient vector flow segmentation of the breast region of interest. WEKA breast data features: 155 × 63 classical features in Weka for TRF, MLP, and BN results. Run “BreastDatasetFeatures.arff” for obtaining the classification results. [file 9807619.f1.zip › 9807619_Addition_SupplementaryMaterials/MATLAB breast data/myImagesSEGMENTEDbreast/2NORMAL/16.bmp]

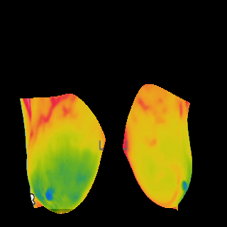

Supplement: Supplementary Materials — Figures 1–7: original images of Figures 1 to 7 in png format. MATLAB breast data: first run “AlexCNNbreast.m” code. Segmentation results of the breast images extracted from the gradient flow method (63 images in bmp format on folder “myImagesSEGMENTEDbreast” divided into normal and abnormal) to feed the convolutional neural network in Matlab2018a (“AlexCNNbreast.m” code to obtain the classification results and CNN models); the two CNN models from the 2-fold cross validation (“myNet_s1.mat” and “myNet_s2.mat”) that obtained 100% of TPR, SPC, and ACC; plotConfMat.m code to obtain the confusion matrices of CNN, TRF, MLP, and BN. Video results of Figures 3 and 4: VIDEO 1 of Figure 4 in mp4 format—this video describes the initial elliptical points for gradient vector flow using the curvature function k of right and left breasts; VIDEO 2 of Figure 4 in mp4 format—this video describes the gradient vector flow segmentation of the breast region of interest. WEKA breast data features: 155 × 63 classical features in Weka for TRF, MLP, and BN results. Run “BreastDatasetFeatures.arff” for obtaining the classification results. [file 9807619.f1.zip › 9807619_Addition_SupplementaryMaterials/MATLAB breast data/myImagesSEGMENTEDbreast/2NORMAL/17.bmp]

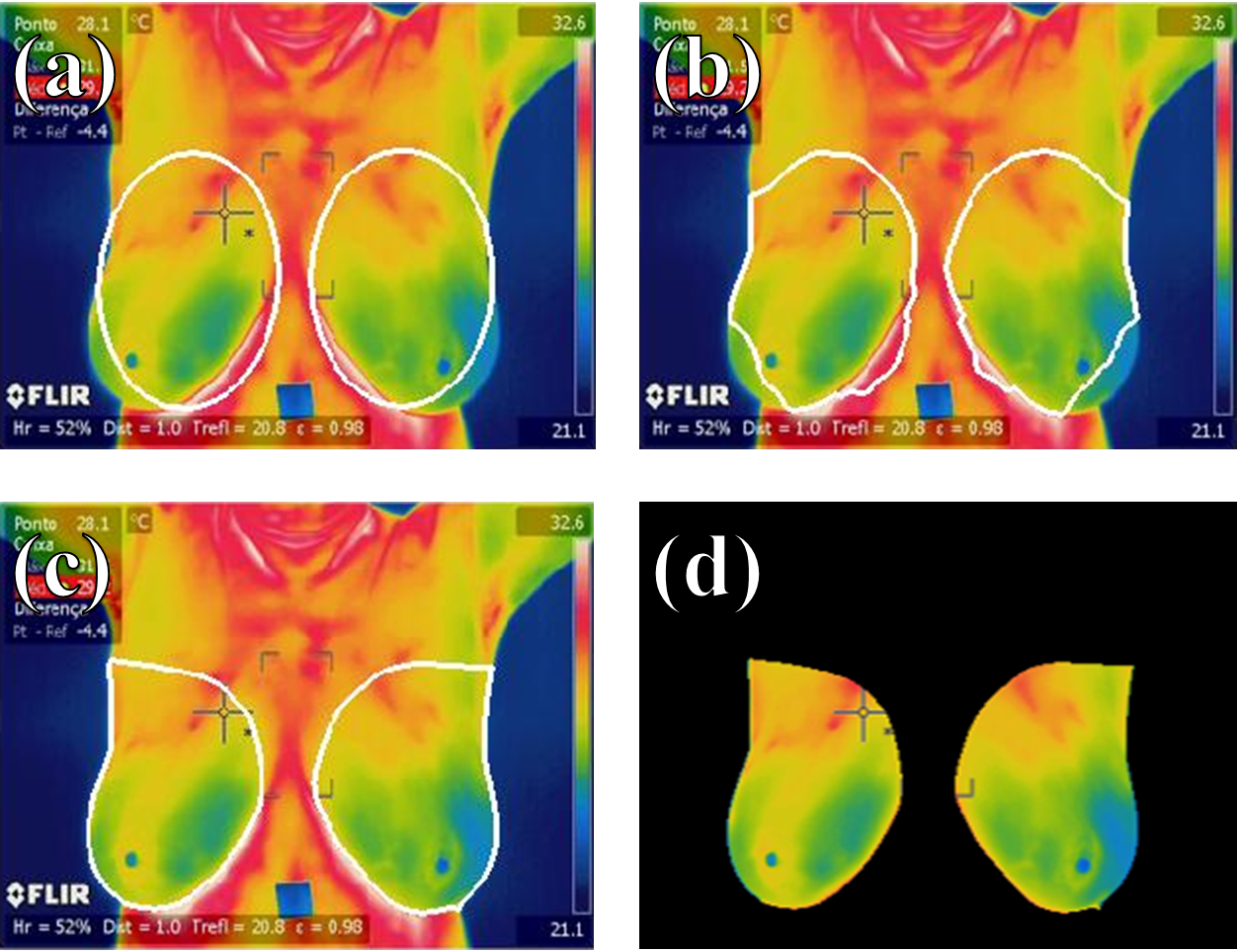

Supplement: Supplementary Materials — Figures 1–7: original images of Figures 1 to 7 in png format. MATLAB breast data: first run “AlexCNNbreast.m” code. Segmentation results of the breast images extracted from the gradient flow method (63 images in bmp format on folder “myImagesSEGMENTEDbreast” divided into normal and abnormal) to feed the convolutional neural network in Matlab2018a (“AlexCNNbreast.m” code to obtain the classification results and CNN models); the two CNN models from the 2-fold cross validation (“myNet_s1.mat” and “myNet_s2.mat”) that obtained 100% of TPR, SPC, and ACC; plotConfMat.m code to obtain the confusion matrices of CNN, TRF, MLP, and BN. Video results of Figures 3 and 4: VIDEO 1 of Figure 4 in mp4 format—this video describes the initial elliptical points for gradient vector flow using the curvature function k of right and left breasts; VIDEO 2 of Figure 4 in mp4 format—this video describes the gradient vector flow segmentation of the breast region of interest. WEKA breast data features: 155 × 63 classical features in Weka for TRF, MLP, and BN results. Run “BreastDatasetFeatures.arff” for obtaining the classification results. [file 9807619.f1.zip › 9807619_Addition_SupplementaryMaterials/Images Figures 1-7/4.png]

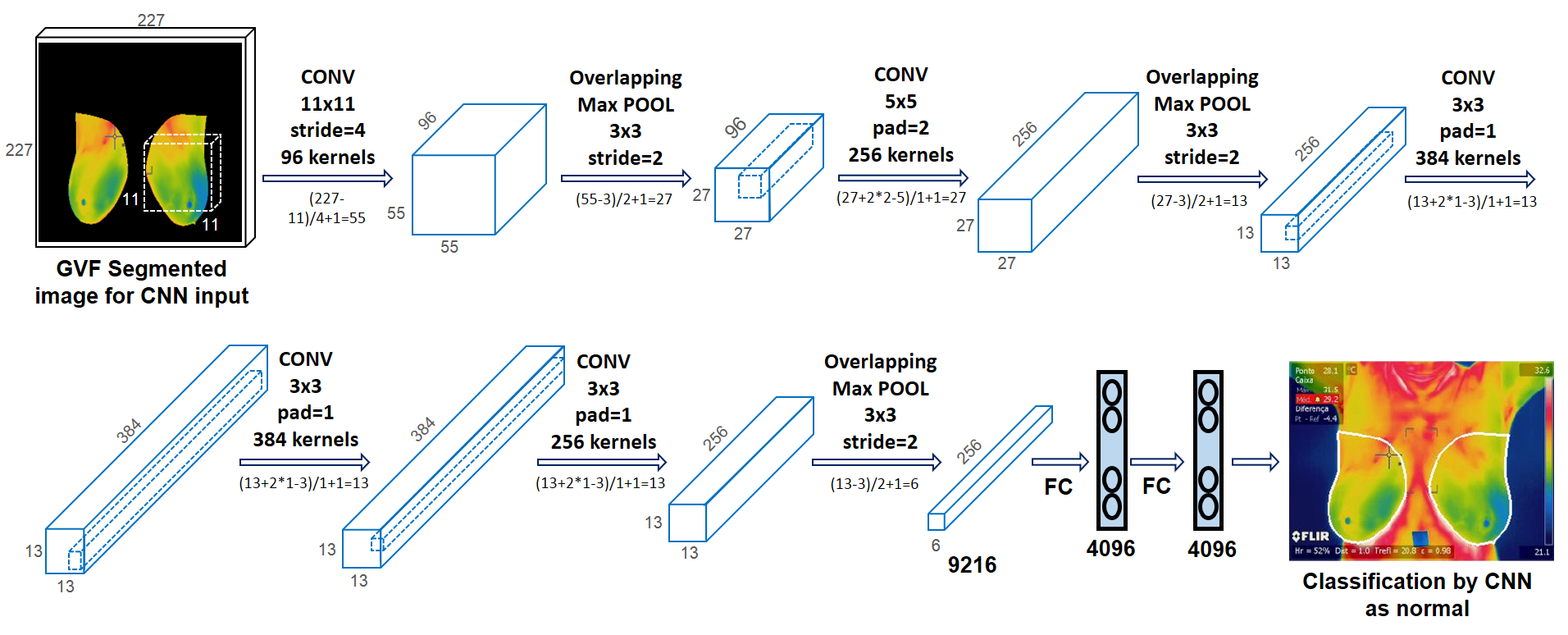

Supplement: Supplementary Materials — Figures 1–7: original images of Figures 1 to 7 in png format. MATLAB breast data: first run “AlexCNNbreast.m” code. Segmentation results of the breast images extracted from the gradient flow method (63 images in bmp format on folder “myImagesSEGMENTEDbreast” divided into normal and abnormal) to feed the convolutional neural network in Matlab2018a (“AlexCNNbreast.m” code to obtain the classification results and CNN models); the two CNN models from the 2-fold cross validation (“myNet_s1.mat” and “myNet_s2.mat”) that obtained 100% of TPR, SPC, and ACC; plotConfMat.m code to obtain the confusion matrices of CNN, TRF, MLP, and BN. Video results of Figures 3 and 4: VIDEO 1 of Figure 4 in mp4 format—this video describes the initial elliptical points for gradient vector flow using the curvature function k of right and left breasts; VIDEO 2 of Figure 4 in mp4 format—this video describes the gradient vector flow segmentation of the breast region of interest. WEKA breast data features: 155 × 63 classical features in Weka for TRF, MLP, and BN results. Run “BreastDatasetFeatures.arff” for obtaining the classification results. [file 9807619.f1.zip › 9807619_Addition_SupplementaryMaterials/Images Figures 1-7/5.png]

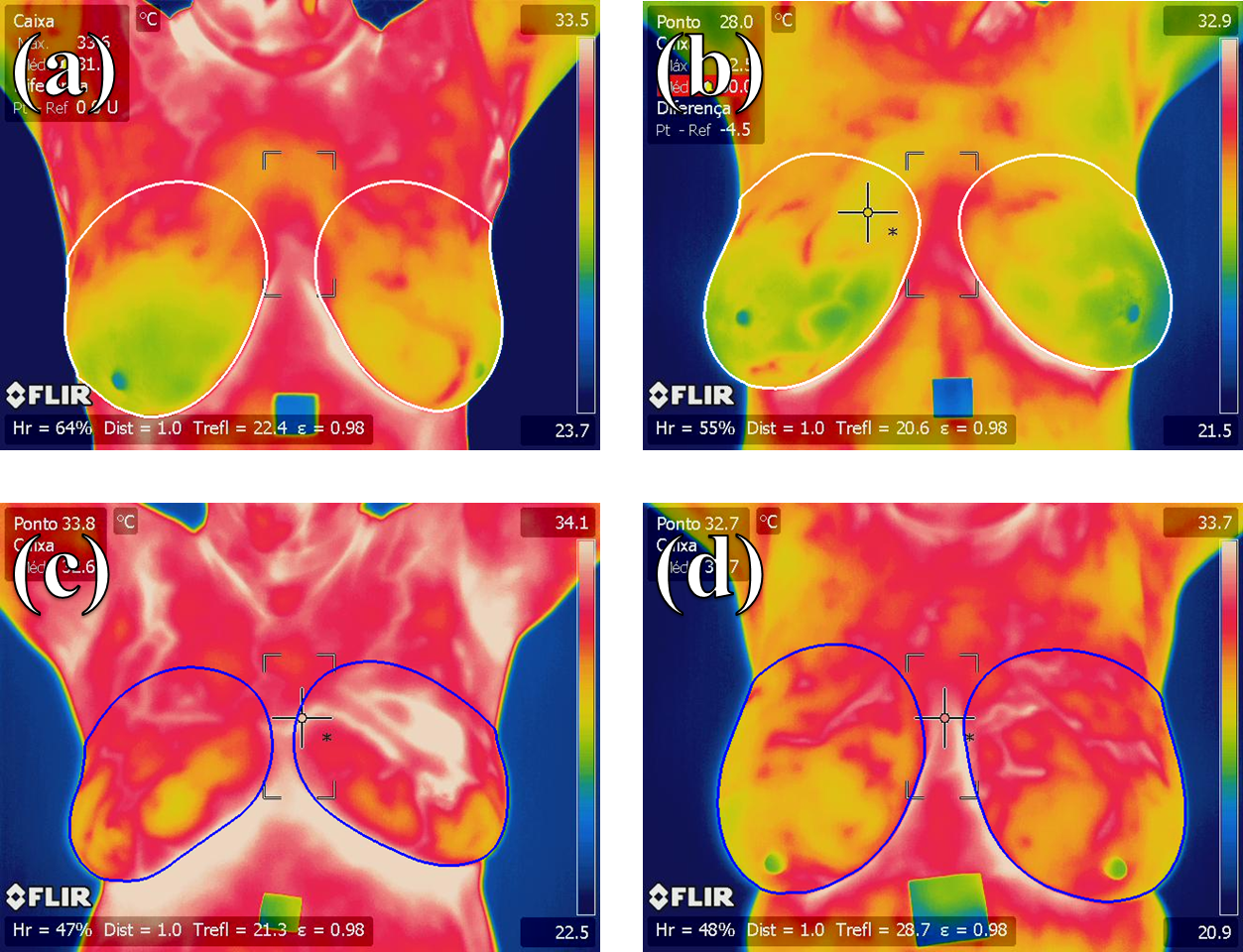

Supplement: Supplementary Materials — Figures 1–7: original images of Figures 1 to 7 in png format. MATLAB breast data: first run “AlexCNNbreast.m” code. Segmentation results of the breast images extracted from the gradient flow method (63 images in bmp format on folder “myImagesSEGMENTEDbreast” divided into normal and abnormal) to feed the convolutional neural network in Matlab2018a (“AlexCNNbreast.m” code to obtain the classification results and CNN models); the two CNN models from the 2-fold cross validation (“myNet_s1.mat” and “myNet_s2.mat”) that obtained 100% of TPR, SPC, and ACC; plotConfMat.m code to obtain the confusion matrices of CNN, TRF, MLP, and BN. Video results of Figures 3 and 4: VIDEO 1 of Figure 4 in mp4 format—this video describes the initial elliptical points for gradient vector flow using the curvature function k of right and left breasts; VIDEO 2 of Figure 4 in mp4 format—this video describes the gradient vector flow segmentation of the breast region of interest. WEKA breast data features: 155 × 63 classical features in Weka for TRF, MLP, and BN results. Run “BreastDatasetFeatures.arff” for obtaining the classification results. [file 9807619.f1.zip › 9807619_Addition_SupplementaryMaterials/Images Figures 1-7/7.png]

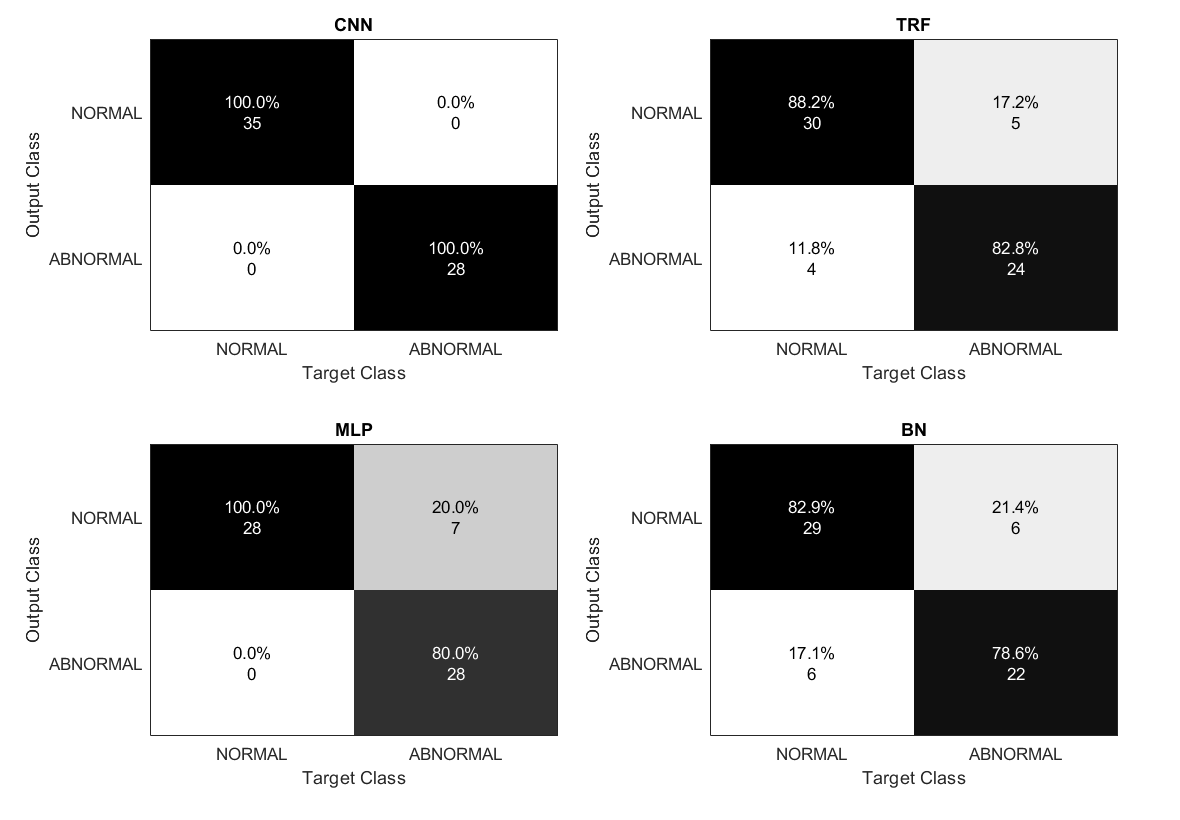

Supplement: Supplementary Materials — Figures 1–7: original images of Figures 1 to 7 in png format. MATLAB breast data: first run “AlexCNNbreast.m” code. Segmentation results of the breast images extracted from the gradient flow method (63 images in bmp format on folder “myImagesSEGMENTEDbreast” divided into normal and abnormal) to feed the convolutional neural network in Matlab2018a (“AlexCNNbreast.m” code to obtain the classification results and CNN models); the two CNN models from the 2-fold cross validation (“myNet_s1.mat” and “myNet_s2.mat”) that obtained 100% of TPR, SPC, and ACC; plotConfMat.m code to obtain the confusion matrices of CNN, TRF, MLP, and BN. Video results of Figures 3 and 4: VIDEO 1 of Figure 4 in mp4 format—this video describes the initial elliptical points for gradient vector flow using the curvature function k of right and left breasts; VIDEO 2 of Figure 4 in mp4 format—this video describes the gradient vector flow segmentation of the breast region of interest. WEKA breast data features: 155 × 63 classical features in Weka for TRF, MLP, and BN results. Run “BreastDatasetFeatures.arff” for obtaining the classification results. [file 9807619.f1.zip › 9807619_Addition_SupplementaryMaterials/Images Figures 1-7/6.png]

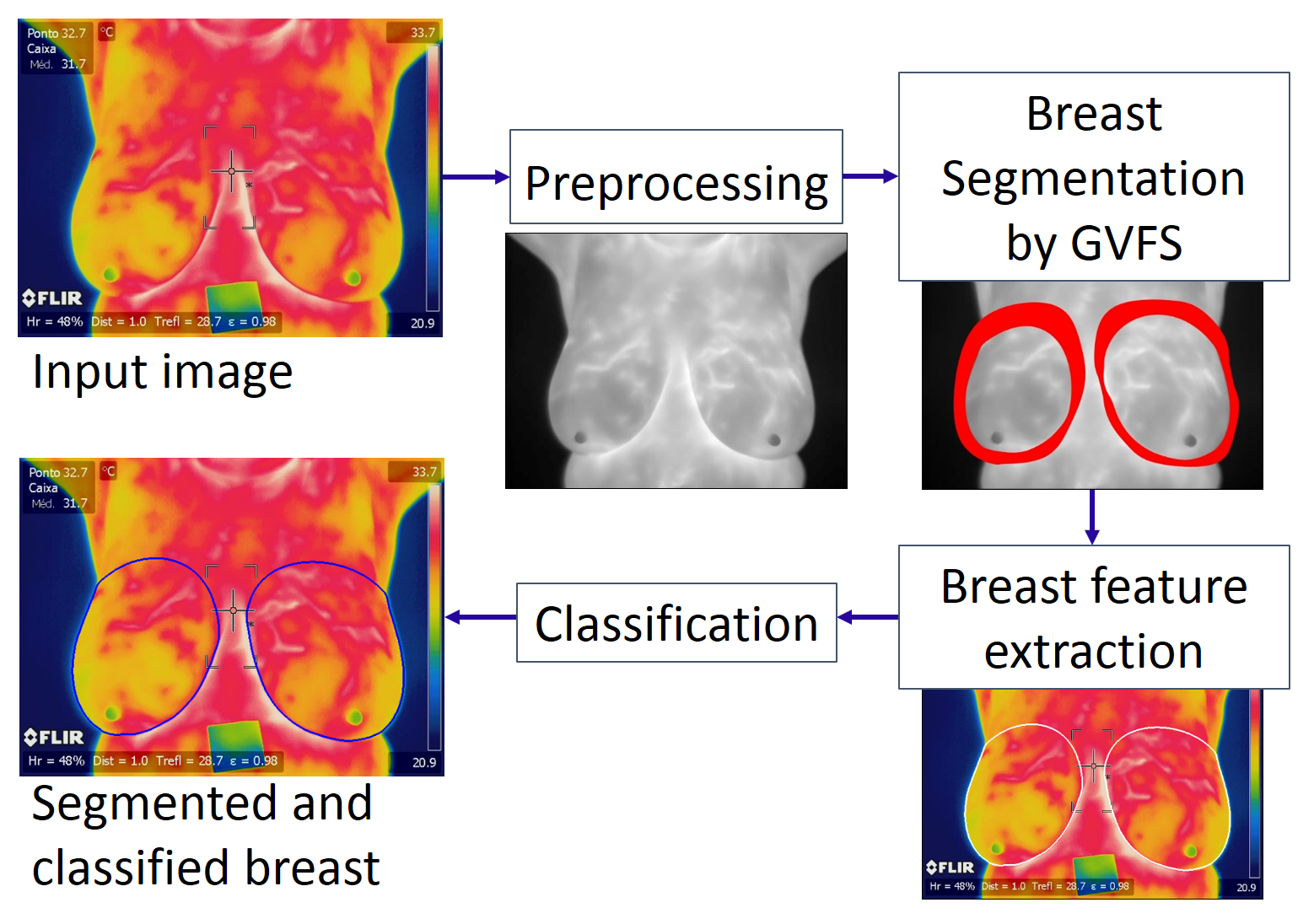

Supplement: Supplementary Materials — Figures 1–7: original images of Figures 1 to 7 in png format. MATLAB breast data: first run “AlexCNNbreast.m” code. Segmentation results of the breast images extracted from the gradient flow method (63 images in bmp format on folder “myImagesSEGMENTEDbreast” divided into normal and abnormal) to feed the convolutional neural network in Matlab2018a (“AlexCNNbreast.m” code to obtain the classification results and CNN models); the two CNN models from the 2-fold cross validation (“myNet_s1.mat” and “myNet_s2.mat”) that obtained 100% of TPR, SPC, and ACC; plotConfMat.m code to obtain the confusion matrices of CNN, TRF, MLP, and BN. Video results of Figures 3 and 4: VIDEO 1 of Figure 4 in mp4 format—this video describes the initial elliptical points for gradient vector flow using the curvature function k of right and left breasts; VIDEO 2 of Figure 4 in mp4 format—this video describes the gradient vector flow segmentation of the breast region of interest. WEKA breast data features: 155 × 63 classical features in Weka for TRF, MLP, and BN results. Run “BreastDatasetFeatures.arff” for obtaining the classification results. [file 9807619.f1.zip › 9807619_Addition_SupplementaryMaterials/Images Figures 1-7/2.png]

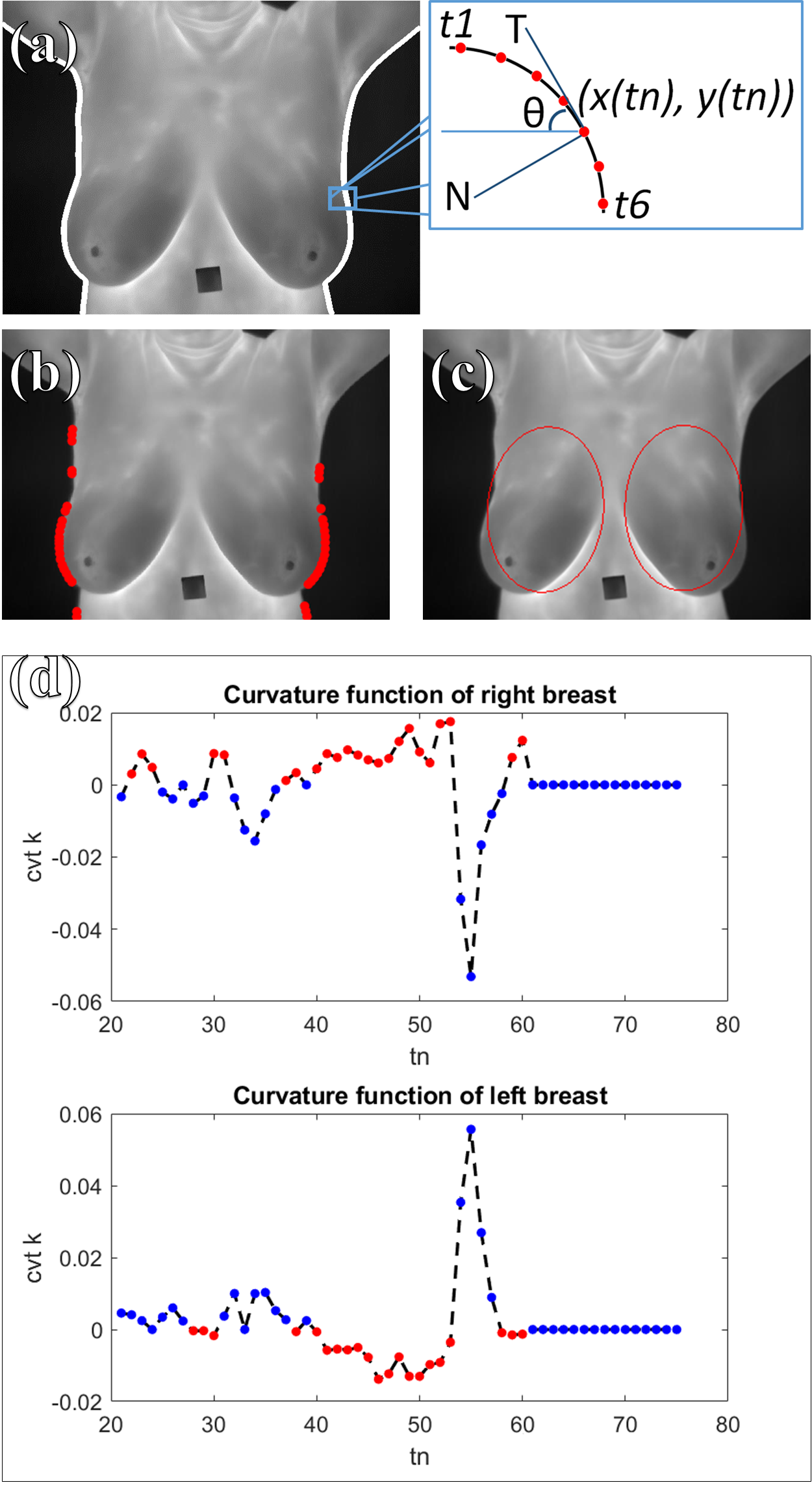

Supplement: Supplementary Materials — Figures 1–7: original images of Figures 1 to 7 in png format. MATLAB breast data: first run “AlexCNNbreast.m” code. Segmentation results of the breast images extracted from the gradient flow method (63 images in bmp format on folder “myImagesSEGMENTEDbreast” divided into normal and abnormal) to feed the convolutional neural network in Matlab2018a (“AlexCNNbreast.m” code to obtain the classification results and CNN models); the two CNN models from the 2-fold cross validation (“myNet_s1.mat” and “myNet_s2.mat”) that obtained 100% of TPR, SPC, and ACC; plotConfMat.m code to obtain the confusion matrices of CNN, TRF, MLP, and BN. Video results of Figures 3 and 4: VIDEO 1 of Figure 4 in mp4 format—this video describes the initial elliptical points for gradient vector flow using the curvature function k of right and left breasts; VIDEO 2 of Figure 4 in mp4 format—this video describes the gradient vector flow segmentation of the breast region of interest. WEKA breast data features: 155 × 63 classical features in Weka for TRF, MLP, and BN results. Run “BreastDatasetFeatures.arff” for obtaining the classification results. [file 9807619.f1.zip › 9807619_Addition_SupplementaryMaterials/Images Figures 1-7/3.png]

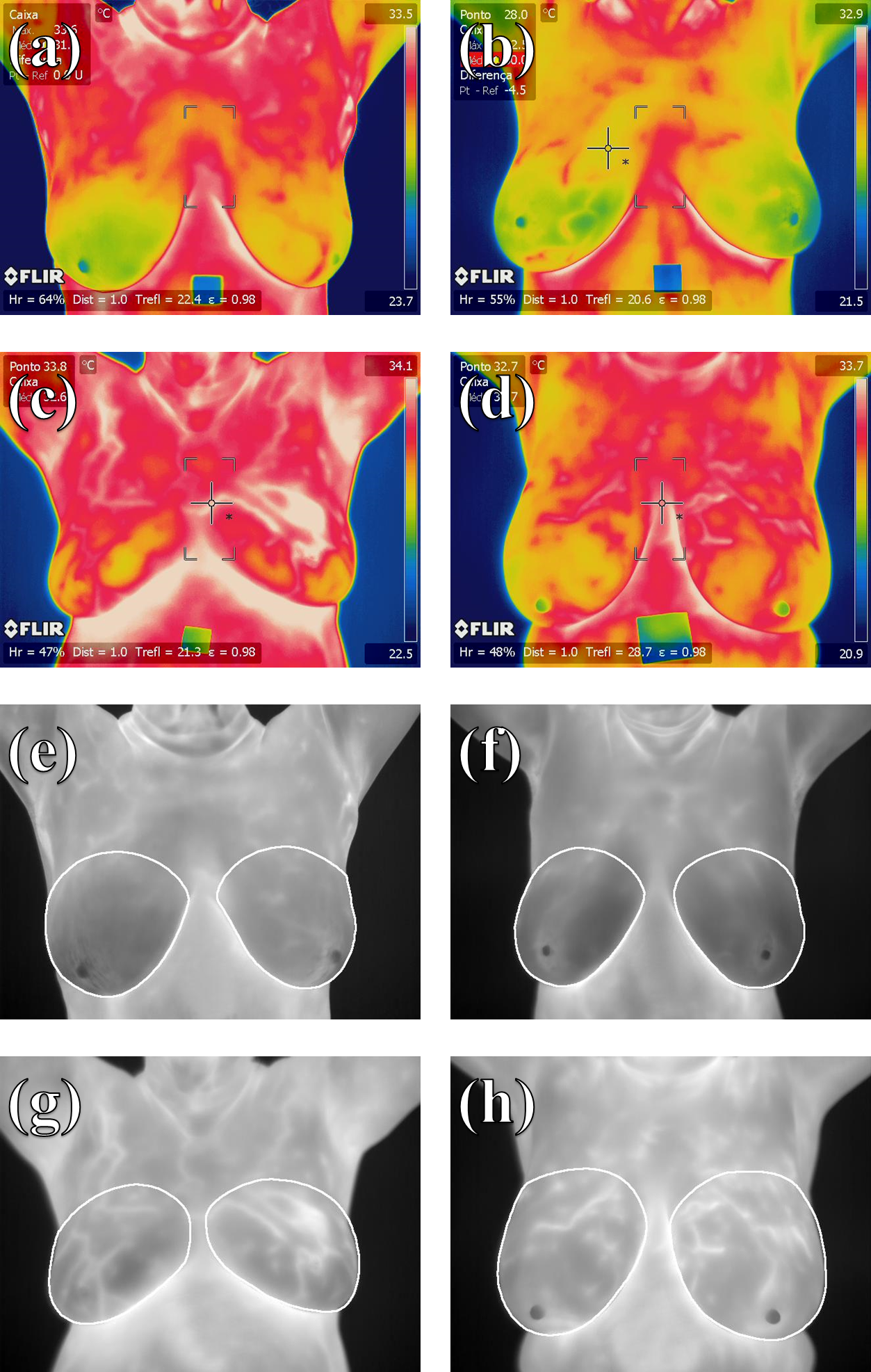

Supplement: Supplementary Materials — Figures 1–7: original images of Figures 1 to 7 in png format. MATLAB breast data: first run “AlexCNNbreast.m” code. Segmentation results of the breast images extracted from the gradient flow method (63 images in bmp format on folder “myImagesSEGMENTEDbreast” divided into normal and abnormal) to feed the convolutional neural network in Matlab2018a (“AlexCNNbreast.m” code to obtain the classification results and CNN models); the two CNN models from the 2-fold cross validation (“myNet_s1.mat” and “myNet_s2.mat”) that obtained 100% of TPR, SPC, and ACC; plotConfMat.m code to obtain the confusion matrices of CNN, TRF, MLP, and BN. Video results of Figures 3 and 4: VIDEO 1 of Figure 4 in mp4 format—this video describes the initial elliptical points for gradient vector flow using the curvature function k of right and left breasts; VIDEO 2 of Figure 4 in mp4 format—this video describes the gradient vector flow segmentation of the breast region of interest. WEKA breast data features: 155 × 63 classical features in Weka for TRF, MLP, and BN results. Run “BreastDatasetFeatures.arff” for obtaining the classification results. [file 9807619.f1.zip › 9807619_Addition_SupplementaryMaterials/Images Figures 1-7/1.png]
